# HIA: a genome mapper using hybrid index-based sequence alignment

**DOI:** 10.1186/s13015-015-0062-4

**Published:** 2015-12-23

**Authors:** Jongpill Choi, Kiejung Park, Seong Beom Cho, Myungguen Chung

**Affiliations:** Division of Bio-Medical Informatics, Center for Genome Science, Korea National Institute of Health, Osong, Korea; Biomedical Research Institute, College of Medicine, Hanyang University, Seoul, Korea

**Keywords:** Hybrid index, NGS, Mapper, Sequence alignment, Hash table index, Suffix array index

## Abstract

**Background:**

A number of alignment tools have been developed to align sequencing reads to the human reference genome. The scale of information from next-generation sequencing (NGS) experiments, however, is increasing rapidly. Recent studies based on NGS technology have routinely produced exome or whole-genome sequences from several hundreds or thousands of samples. To accommodate the increasing need of analyzing very large NGS data sets, it is necessary to develop faster, more sensitive and accurate mapping tools.

**Results:**

HIA uses two indices, a hash table index and a suffix array index. The hash table performs direct lookup of a q-gram, and the suffix array performs very fast lookup of variable-length strings by exploiting binary search. We observed that combining hash table and suffix array (hybrid index) is much faster than the suffix array method for finding a substring in the reference sequence. Here, we defined the matching region (MR) is a longest common substring between a reference and a read. And, we also defined the candidate alignment regions (CARs) as a list of MRs that is close to each other. The hybrid index is used to find candidate alignment regions (CARs) between a reference and a read. We found that aligning only the unmatched regions in the CAR is much faster than aligning the whole CAR. In benchmark analysis, HIA outperformed in mapping speed compared with the other aligners, without significant loss of mapping accuracy.

**Conclusions:**

Our experiments show that the hybrid of hash table and suffix array is useful in terms of speed for mapping NGS sequencing reads to the human reference genome sequence. In conclusion, our tool is appropriate for aligning massive data sets generated by NGS sequencing.

**Electronic supplementary material:**

The online version of this article (doi:10.1186/s13015-015-0062-4) contains supplementary material, which is available to authorized users.

## Background

Recent studies based on next-generation sequencing (NGS) technology have produced hundreds or thousands of exome or whole genome sequences with decreasing cost of NGS experiments [1]. As the NGS technologies evolve, NGS technologies have gradually increased read length and decreased error rate [2]. To keep pace with developing NGS technologies, many alignment tools have been developed for both short and long reads. These tools include SSAHA2 [3], BWA [4, 5], AGILE [6], SOAP2 [7], Bowtie2 [8], SeqAlto [9] and others. Among them, many aligning programs use index-based mapping strategy. For example, SSAHA2, AGILE and SeqAlto use a hash table (HT) index of a reference genome, whereas BWA, SOAP2 and Bowtie2 use an index of the reference genome based on the Burrows–Wheeler transform [10].

All HT-based alignment tools use the same seed-and-extend strategy [11, 12], which proceeds by searching for candidate alignment regions (CARs), aligning each location, and reporting the best alignments. A HT index supports very fast lookup of candidate locations with q-grams (strings of length q). Smaller q increases the sensitivity and the number of CARs, whereas larger q decreases the sensitivity and number of CARs. Furthermore, because q is fixed, the HT must be rebuilt when q-grams of a new length are needed. Most BWT-based alignment tools use the full-text minute index [13], which is memory-efficient and similar to the suffix tree. With respect to matching time, the suffix tree is efficient for exact matching, although it is slow for inexact matching. BWA and Bowtie2 follow similar seed-and-extend approaches, including the use of HT-based algorithms for long reads.

Support of long-read alignment, high speed, accuracy, and sensitivity are essential features of the current NGS mapping tools. Here, we tried to merge the advantages of HT-based and suffix tree-based alignment in a tool that satisfies these requirements. To this end, we developed a genome mapper using hybrid index-based sequence alignment (HIA).

In this article, we describe the HIA tool, and show the results of comparisons of performance on simulated and real read data between HIA and the other four popular alignment tools including BWA, Bowtie2, SOAP2 and SeqAlto. The results of the benchmark analysis demonstrate that HIA outperforms the other aligners, especially in speed.

## Methods

### Hybrid index

Let Σ be an alphabet and S = s_0_s_1_ … s_m−1_ be a string over Σ. Let |S| = m be the length of S, S[i] = s_i_ be the i-th symbol of S, S[i, j] = s_i_ … s_j_ be a substring, and S_i_ = S[i,m−1] be a suffix of S. We define a q-gram as a substring of S with length q. In the context of DNA sequence, the alphabet Σ consists of the four nucleotides A, C, G, and T, i.e., Σ = {A, C, G, T}. We assign A, C, G, and T to the numbers 0, 1, 2, and 3, respectively. Thus, each q-gram is encoded as an unsigned integer with two bits per nucleotide. However, most reference genome sequences contain a nucleotide other than {A, C, G, T}, such as ‘N’. This occasionally happens with NGS read sequences as well. We replace ‘N’ with a uniform random nucleotide such as BWA and many other tools do.

**Fig. 1:**
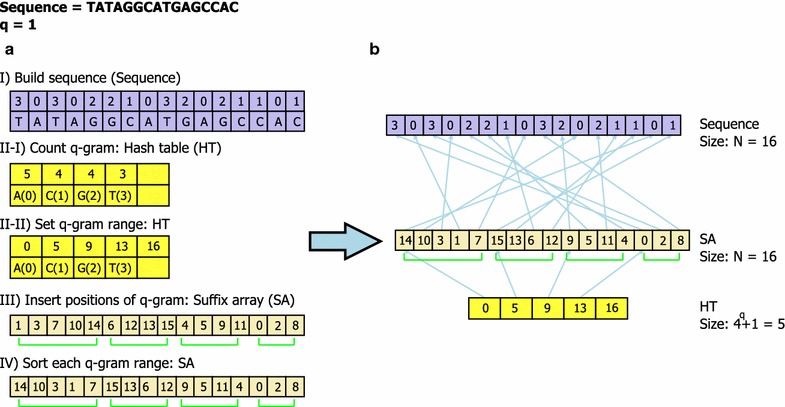
Constructing the hybrid index. *Panel* **a** represents the procedure for constructing the hybrid index given Sequence = TATAGGCATGAGCCAC and q = 1. Construction proceeds as follows: first, convert the nucleotide symbols in sequence into the corresponding decimal values (*I*). Second, count each q-gram and store the counts in the HT (*II*-*I*). Third, set the beginning position of each q-gram based on the counts of q-grams (*II*-*II*). Fourth, store the positions of each q-gram in the SA such as (*III*). Finally, sort each q-gram range in the SA and finish hybrid index construction. The sizes of Sequence, SA, and HT are 16, 16, and 4^q^ + 1=5, respectively. *Panel* **b** shows the constructed hybrid index

In terms of a hybrid index, SOAP2 implements a hash table on the FM-index which is a compressed SA. On the other hand, our hybrid index consists of a reference sequence, a suffix array (SA), and a hash table (HT), as in Fig. 1. Since there are four symbols in the alphabet, a reference sequence of length N can be packed into N/4 bytes. The SA is an array of the starting positions (integers) of suffixes of the reference sequence in lexicographical order. The number of suffixes of a sequence of size N is N. The HT is an array of pointers into SA indicating which positions in SA belong to which q-grams. Since we define a q-gram as a string of length q, the number of elements of HT is 4^q^ + 1. Given a q-gram, HT[*x*] is the first position of the q-gram in SA, where *x* is the numeric value of the q-gram. We define the range (R) of the q-gram in SA by (1):

1 $$R(x) = [{\text{HT}}[x],\;{\text{HT}}[x + 1] - 1]$$

If the q-gram does not exist in the sequence, HT[*x*] is the first position of next existing q-gram in the sequence so that HT[*x*] and HT[*x* + 1] are the same, and R(*x*) is empty.

The procedure for constructing the hybrid index consists of four processes, as follows: (1) packing the reference sequence in a 2-bits per base format (Sequence); (2) counting each q-gram in the sequence and set the range of q-grams in SA (HT); (3) inserting the sequence positions of each q-gram into its range of SA; and (4) sorting the range for each q-gram (SA) lexicographically. Based on the heuristic determining the final positions of most of the suffixes using only the first few symbols of each suffix [14], the SA construction algorithm proceeds sorting the length-w prefixes of the suffixes in a q-gram range. If some suffixes share also a length-w prefix, then the sort is repeated by sorting the length-w substrings that follow the length-w prefixes, and so on. In order to reduce the sequence access time, length-w prefixes are converted to integer values. In the case that the size of the memory word is 4 bytes and the size of the alphabet is 4, the w is set to between 0 and 16. The ranges of q-grams are not overlapped so that the fourth process can also be parallelized. Figure 1 describes the underlying data structure and the method for constructing a hybrid index of a reference sequence.

Retrieving positions of a query Q in the sequence is implemented in two steps: HT lookup and binary search of the SA. If the prefix of Q of length q (q-gram) is a substring of the sequence, we find the range (R) in which the q-gram belongs in the SA, using Eq. (1). Otherwise, the range (R) returns an empty range, indicating that Q is not in the sequence. If the returned range is not empty, we next find the positions at which Q occurs in the sequence by binary search of the substring Q[q, |Q| − 1], based on the SA. Theoretically, searching a length-m substring in a string of length N by SA can be implemented in O(mlogN) time in the worst case. The hash table index can reduce the length of searching string such as (m′ = m − q) and reduce the size of the searching range such as (n′ ≪ N). When the reference sequence is the GRCH37 build of the human genome and q is 14, the length of packed sequence is 2,861,343,766 and the average size of the searching range is 14.12. Our experiment showed that the hash table index can decrease considerably the searching time (see Additional file 1).

### Hybrid mapping: finding candidate alignment region (CAR)

Hybrid mapping follows the same seed-and-extend approach used by all HT-based tools. A MR (matching region) is a common substring between the reference sequence and the read. Let ‘sp’ be the starting position in the reference sequence where the MR occurs. We will indicate each MR as 3-tuple <dv, ro, L>, where ‘ro’ (read offset) is the starting position in the read where the MR occurs, ‘dv’ (diagonal value) is defined as dv = sp − ro, and ‘L’ is the length of the MR. Given a length-m′ MR, there are (m′ − q + 1) q-grams having the same diagonal values and consecutive read offsets. Diagonal values having same values implies that the corresponding MRs are close each other in the reference sequence. A CAR (candidate alignment region) is a list of MRs, which are close each other and ordered by ‘ro’. We define a CAR as a seed and align only the unmatched regions in CAR.

The procedure for finding MRs and CARs of a read is as follows: (1) retrieving range of SA of each q-gram using HT and SA; (2) computing diagonal values; (3) sorting by diagonal value and offset; (4) grouping MRs with same diagonal value and successive offsets; (5) merging the adjacent MRs into CARs; (6) sorting the CARs by matched bases in descending order. For example, given a read (r = GCCATG) and q-gram length (q = 2) and the hybrid index constructed in Fig. 1, we can find MRs and CARs as follows:

I. Retrieve range of SA of each q-gram using HT and SA

0-th q-gram positions: (GC: SA[10, 11])

1-th q-gram positions: (CC: SA[8])

2-th q-gram positions: (CA: SA[6, 7])

3-th q-gram positions: (AT: SA[3, 4])

4-th q-gram positions: (TG: SA[15])

II. Compute diagonal values

(GC, 5, 0), (GC, 11, 0), (CC, 11, 1), (CA, 4, 2), (CA, 11, 2), (AT, −2, 3), (AT, 4, 3), (TG, 4, 4)

III. Sort by diagonal value and offset

(AT, −2, 3), (CA, 4, 2), (AT, 4, 3), (TG, 4, 4), (GC, 5, 0), (GC, 11, 0), (CC, 11, 1), (CA, 11, 2)

IV. Group MRs with same diagonal value and successive offsets

MR0: (AT, −2, 3, 2) ← (AT, −2, 3)

MR1: (CATG, 4, 2, 4) ← (CA, 4, 2), (AT, 4, 3), (TG, 4, 4)

MR2: (GC, 5, 0, 2) ← (GC, 5, 0)

MR3: (GCCA, 11, 0, 4) ← (GC, 11, 0), (CC, 11, 1), (CA, 11, 2)

V. Merge the adjacent MRs into CARs and set matched bases

CAR0: (MR0; 2)

CAR1: (MR1, MR2; 5)

CAR2: (MR3; 4)

VI. Sort the CARs by matched bases

CAR1: (MR1, MR2; 5)

CAR0: (MR3; 2)

CAR2: (MR2; 2).

Although diagonal values of two adjacent MRs are different, they could be located in a same CAR if there were inserted or deleted bases between them. In the case of CAR1, the difference value between diagonal value of MR1 and diagonal value of MR2 is 1 and there is one inserted base (C) between MR1 and MR2. We refer to this value as adjacency and we use the value in order to set the permitted size of insertion and deletion between MRs.

In order to find MRs and CARs efficiently, we apply three heuristics. Let the read length and the error rate be m and ε, respectively. The first heuristic is that there is a common substring of length at least m/(k + 1) between two reads of length m with k differences [15]. Let λ = εm be the expected number of errors in a read and let X be the random variable. We can compute the chance of observing a read with at most k errors as below.

2 $${\text{P}}\left\{ {{\text{X}} \le {\text{k}}} \right\} = \sum\limits_{{{\text{i}} = 0}}^{{\text{k}}} {{\text{e}}^{{ - \lambda }} \lambda ^{{\text{i}}} } /{\text{i}}!$$

The formula (2) is the cumulative distribution function of X. We are able to calculate the rate of reads with at most k errors according to the formula (2). If we set k = 1.5λ, the rate of reads with at most 1.5λ errors approach to 0.9 and we can use the q-gram with length m/(1.5λ + 1). Using a q-gram with the same length as the common substring decreases the number of MRs and CARs.

Secondly, since a q-gram that occurs in many regions in the sequence is not a good discriminator, such q-gram was given less weight than one that occurs in few regions. This heuristic is based on the inverse document frequency (IDF) commonly used in the field of information retrieval. The IDF is a measure of whether or not the term is common across all documents [16]. Applying this heuristic, we can filter out the less weight q-grams and consequently skip undesirable MRs and CARs.

Finally, given a read (r) and two strings, S1 and S2, both of length m, if the number of matched bases between r and S1 is greater than the number of matched bases between r and S2, then the number of differences between r and S1 is smaller than the number of differences between r and S2. This heuristic can be used to rank CARs by the number of matched bases and to filter out the lower-ranked CARs.

### Hybrid mapping: aligning candidate alignment region (CAR)

**Fig. 2:**
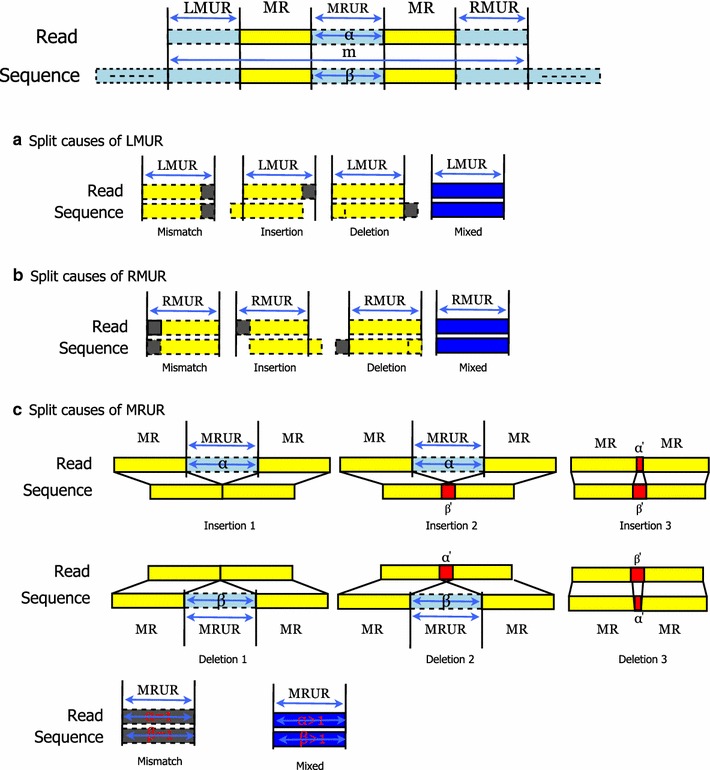
Classification diagram of split causes between matched region and unmatched region. Panel **a** represents the split causes of LMUR. Panel **b** shows the split cuases of RMUR. There are four split causes (Mismatch, Insertion, Deletion, and Mixed) in LMUR and RMUR. Panel **c** represents the split causes of MRUR, that can be classified in great detail. *Yellow block* indicates that both blocks of read and sequence are exactly matched. *Dark gray block* means the mismatch region. *White block* shows that two blocks are gapped. *Red block* shows that two blocks are overlapped. Finally, *blue block* indicates that there are two more split causes on read block and sequence block

To align the top-ranked CARs, we simply align the unmatched regions in each CAR, because the MRs are already aligned (matched). We classify the unmatched regions into three groups: leftmost unmatched region (LMUR), which is the left unmatched region of the first MR in a CAR; rightmost unmatched region (RMUR), which is the right unmatched region of the last MR in a CAR; and unmatched regions between two MRs (MRURs). The matched and unmatched region can be separated because of mismatch, insertion and deletion. We analyze these split causes such as Fig. 2. In the cases of LMUR and RMUR, if the only adjacent bases between these unmatched regions and MR are mismatched and other bases are matched then the split cause is Mismatch; if they are inserted and others are matched then the split cause is Insertion; if they are deleted and others are matched then the split cause is Deletion; otherwise the split cause is Mixed. The Mismatch, Insertion and Deletion information clearly indicate how these unmatched regions are aligned. Thus, we can apply the Needleman–Wunsch algorithm [17] only to the mixed cause unmatched region so that we can reduce computation time for the dynamic programming method of the Needleman–Wunsch algorithm.

**Fig. 3:**
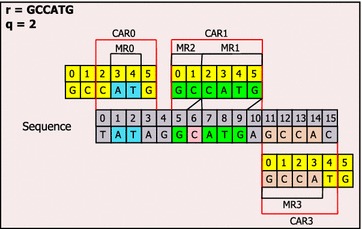
Finding MRs and CARs. Find MRs and CARs given a read (r = GCCATG) and the hybrid index constructed in Fig. 1

There are also many split causes of MRUR like as Fig. 2. They can be categorized into Mismatch, Insertion, Deletion, and Mixed. As regards Insertion, if there were some bases (α) between two adjacent MRs in a read but there were no base in the sequence then α bases could be inserted (Insertion 1); if there were α bases between two adjacent MRs in a read and β bases were overlapped in the sequence then α + β bases could be inserted (Insertion 2); if α bases were overlapped in a read and β bases were overlapped in the sequence and α were smaller than β then β − α bases could be inserted (Insertion 3). With respect to Deletion, if there were β bases between two adjacent MRs in the sequence but there were no base in a read then β bases could be deleted (Deletion 1); if there were β bases between two adjacent MRs in the sequence and α bases were overlapped in the sequence then α + β bases could be deleted (Deletion 2); if α bases were overlapped in a read and β bases were overlapped in the sequence and α were greater than β then α − β bases could be deleted (Deletion 3). As to Mismatch, if there were one base between two adjacent MRs in a read and there were one base in the sequence then these bases could be mismatched (Mismatch). Except all the causes mentioned above, the others are Mixed. We also can apply the Needleman–Wunsch algorithm only to the Mixed cause MRUR in order to reduce the dynamic programming load. For example, in the case of CAR1 in Fig. 3, there is one MRUR between MR1 and MR2. Through “Insertion 2”, we know one insertion between MR1 and MR2, and then obtain 2M1I3M (CIGAR format). In the case of CAR 2, there is one RMUR and the split cause is “Mixed.” Thus we can apply Needleman-Wunsch to the RMUR, and then obtain 4M1I1M or 5M1I (CIGAR format).

### Extended hybrid mapping

Hybrid mapping might miss some CARs and result in the failure of mapping (unmapped). The main reasons for missed CARs might be one of following reasons: (1) too many errors in a read; (2) too many highly frequent q-grams in a read; and (3) many high-ranked CARs. In the first case, a long q-gram based on the first heuristic approach results in empty q-gram ranges. In the second case, the second heuristic approach causes highly frequent informative q-grams to be missed. In the third case, the lower-ranked but informative CARs are lost because of the third heuristic approach. We applied extended hybrid mapping to the unmapped, which uses shorter q-grams including some highly frequent q-grams, and extends ranked CARs further. Together, these modifications increase the sensitivity of the technique.

### Implementation

We implemented HIA on Java to support multiple platforms (see Additional file 2). HIA takes the reference sequence FASTA file as the input, builds the hybrid index, and then outputs the hybrid index: SA file (.sa), HT file (.idx), packed sequence file (.seq), and reference sequence information file (.seqInfo). For the alignment, HIA takes the hybrid index and a query FASTQ file as inputs, and outputs the mapped and unmapped alignments in SAM format. To reduce the bottle-neck on reading reads and writing mapped results, we divided the alignment procedure into reading, mapping, and writing procedures. Each procedure runs in an independent thread and is scheduled using three queues. Furthermore, HIA also outputs a report, consisting of a summary file of mapping results and two pie chart graphs of the mapping rate, to inform the user about the mapping results. Additionally, during the alignment, HIA summarizes the FASTQ input and reports the basic statistics and base quality information: statistics of FASTQ; base quality per read position, as a bar graph; base quality, as a heat map; and quality score, as a box-plot graph. This summary is useful to determine the quality of the NGS sequence data that is produced. We used JFreeChart [18] to generate all graphs.

## Results and discussion

### Evaluation data sets and evaluation measurements

We made six datasets from the GRCH37 build of the human genome, using Mason [19]. Two of these are unpaired Illumina-like datasets, consisting respectively of one million 100 bp reads and one million 150 bp reads, which Mason simulated with parameters ‘illumina -hn 2 -sq -n 100 -N 1000000’ and ‘illumina -hn 2 -sq -n 150 -N 1000000’. The next two datasets are paired Illumina-like read datasets, which Mason simulated with parameters ‘illumina -hn 2 -sq -rn 2 -mp -ll 375 -le 100 -n 100 -N 1000000’ and ‘illumina -hn 2 -sq -rn 2 -mp -ll 375 -le 100 -n 150 -N 1000000’. The last two datasets are unpaired 454-like read datasets, which Mason simulated with parameters ‘454 -hn 2 -sq -rn 2 -k 0.3 -bm 0.4 -bs 0.2 -nm 250 -N 1000000’ and ‘454 -hn 2 -sq rn 2 -k 0.3 -bm 0.4 -bs 0.2 -nm 400 -N 1000000’. Mason also generated the correct alignment results of the six read datasets in SAM format. The exact command-line parameters and descriptions for each dataset can be found in the Additional file 1.

To assess the performance on real data, we obtained the Illumina dataset from a human re-sequencing study [20] and the 454 dataset from the 1000 Genomes Project Pilot (1000 Genomes Project Consortium, 2010). The Illumina dataset consists of 1,296,188,286 101 bp × 99 bp paired-end reads. The 454 dataset has NCBI Short Read Archive accession number SRR003161 and contains 1,375,489 reads with an average length of 355 bp. We made three test datasets from the Illumina dataset and one test dataset from the 454 dataset such as (1) one million paired-end HiSeq reads, (2) one million 101 bp single-end HiSeq reads, (3) the whole of the paired-end HiSeq reads, and (4) the whole of the 454 reads.

We applied the following four evaluation measures in benchmark study: Aligned (%), Unique (%), Q10 (%) and Time (s). The Aligned (%) denotes the percent of aligned reads over total reads and indicates the overall mapping rate. The Unique (%) measures the percent of uniquely aligned reads over total reads and refers to MAPQ ≥ 1. The Q10 (%) measures the fraction of mapped reads MAPQ ≥ 10. The Time is the elapsed time (seconds) including both the index loading time and the alignment time. In the case of the simulated datasets, the %Err measures the percent of wrong aligned reads over the reads satisfying Unique (%) or Q10 (%). We adopt the concept of a correct alignment from Langmead and Salzberg [8], who determined an alignment correct only if the alignment was on the same strand and the leftmost position was within 50 bp of the assigned position.

### Evaluation results

To evaluate the performance of HIA, we compared HIA to BWA, Bowtie2, SOAP2 and SeqAlto on six simulated datasets and two real datasets. In all tests, we used the GRCH37 build of the human genome as the reference sequence used in alignment. We performed the alignments using a computer with two Intel Xeon 6-Core X5670 2.93-GHz processors and 48 GB RAM. All alignment tools were run with a single thread for alignment except for multi-thread tests.

#### Performance of index generation

Table 1 shows the results for index generation. These results indicate that the indexing time of HIA is comparable to other aligners. Especially, HIA is able to reduce the time of index generation by using multiple threads of modern multi-core computers (Additional file 1).

**Table 1:** Results of index generation

Aligner	Options	Time	Memory (GB)	Size (GB)
HIA	-t 1 -q 14	165	20.32	12.63
HIA	-t 12 -q 14	28	20.47	12.63
BWA		65	4.53	5.40
bowtie2		99	5.35	4.10
soap2		55	3.39	5.90
seqAlto	-I 0 genome.fa 28	33	37.99	22.40
seqAlto	-I 1 genome.fa 22	12	13.19	5.52

Time measurement is elapsed time (minute). Memory is the peak memory for the index construction. Size is the sum of all generated files

In the perspective of SA construction, we performed several tests of index generation and compared the results from our index generation algorithm and the divsufsort that is one of the best SA construction algorithms [21]. It was clear that the divsufsort outperforms our algorithm in building the SA of the human genome (see Additional file 1). We bypassed this problem through implementation of the multiple threading in the construction of the SA of the human genome (see comparison of the performance of the multiple threading and divsufort algorithm in Additional file 1). Moreover, since the construction of the SA is required once in the alignment of the NGS data, we believe that this would be not serious problem in practical application.

#### Results of simulated single-end reads

We ran six of the aligners with various parameter settings for 100, 150, 250 and 400 bp single-end reads. All results can be found in the Additional file 1. SeqAlto can only align Illumina-like reads, so it was excluded from the tests for 454-like datasets.

Table 2 shows the best results from the sensitivity and precision perspectives. For both Illumina-like datasets and 454-like datasets, HIA is significantly faster than all of the other aligners except BWA MEM and SOAP2. SOAP2 is very fast, but not as sensitive as HIA. BWA is slightly more accurate, but not as sensitive as HIA for Illumina-like datasets. However, HIA is more sensitive and accurate than BWA for 454-like datasets. Bowtie2 is similar to HIA with regard to sensitivity, but not as accurate as HIA for both Illumina-like datasets and 454-like datasets. SeqAlto is slightly more accurate, but not as sensitive as HIA for Illumina-like datasets. BWA MEM is more accurate and more sensitive than HIA for Illumina-like datasets, but not as sensitive as HIA for 454-like datasets.

**Table 2:** Results for simulated single-end reads

Aligner	Time	% Aligned	% Unique [% Err]	% Q10 [% Err]
(a) Illumina-like 100 bp reads (unpaired)				
HIA	464	100.00	96.57 [0.4314]	95.80 [0.2151]
BWA	1242	98.11	94.73 [0.1711]	94.60 [0.1562]
BWA MEM	265	100.00	96.30 [0.0497]	95.27 [0.0153]
Bowtie2	1291	99.95	99.63 [2.4252]	94.22 [0.0208]
SOAP2	264	79.37	76.27 [0.4679]	
SeqAlto	1459	99.69	96.33 [0.2861]	96.04 [0.2156]
(b) Illumina-like 150 bp reads (unpaired)				
HIA	530	100.00	97.56 [0.2552]	97.26 [0.1643]
BWA	2464	98.00	95.55 [0.0953]	95.48 [0.0866]
BWA MEM	355	100.00	97.36 [0.0210]	96.41 [0.0053]
Bowtie2	2069	99.97	99.87 [1.6663]	95.99 [0.0094]
SOAP2	525	68.72	66.78 [0.2806]	
SeqAlto	3608	99.68	97.25 [0.1947]	97.10 [0.1490]
(c) 454-like 250 bp reads (unpaired)				
HIA	1009	99.96	98.28 [0.4189]	94.38 [0.1772]
BWA-SW	3157	99.86	97.61 [0.6735]	94.38 [0.0357]
BWA MEM	1497	100.00	97.92 [0.0767]	97.26 [0.0346]
Bowtie2	2947	99.59	83.40 [0.5980]	36.44 [0.0011]
(d) 454-like 400 bp reads (unpaired)				
HIA	1378	99.76	98.48 [0.1557]	96.17 [0.0397]
BWA-SW	5144	100.00	95.89 [0.2084]	94.00 [0.0284]
BWA MEM	2426	99.99	98.46 [0.0471]	97.98 [0.0238]
Bowtie2	6597	99.96	88.35 [0.3048]	32.93 [0.0000]

Time measurement is elapsed time (second). Unique refers to MAPQ ≥ 1 if MAPQ available. Q10 refers to MAPQ ≥ 10

#### Results of simulated paired-end reads

We also ran six of the aligners with the same parameter settings as in the single-end reads for 100 and 150 bp paired-end reads. All results can be found in the Additional file 1.

Table 3 shows the best results from the sensitivity and precision perspectives. For both datasets, HIA is significantly faster than all of the other aligners except BWA MEM and SOAP2 while retaining good alignment sensitivity and precision. SOAP2 is very fast, but not as sensitive as HIA. BWA and SeqAlto are similar to HIA with respect to sensitivity. BWA MEM is more accurate than the other aligners. Bowtie2 is less sensitive as compared with HIA, BWA and SeqAlto.

**Table 3:** Results for simulated paired-end reads

Aligner	Time	% Aligned	% Unique [% Err]	% Q10 [% Err]
(a) Illumina-like 100 bp reads (paired)				
HIA	1009	99.96	97.17 [0.0859]	96.75 [0.0510]
BWA	2554	99.79	97.70 [0.0954]	97.49 [0.0692]
BWA MEM	646	99.99	98.03 [0.0282]	97.95 [0.0172]
Bowtie2	1691	97.13	97.09 [1.2711]	93.73 [0.0128]
SOAP2	586	84.54	82.75 [0.3356]	
SeqAlto	2945	99.61	97.15 [0.0833]	97.02 [0.0788]
(b) Illumina-like 150 bp reads (paired)				
HIA	1153	99.99	98.10 [0.0780]	97.89 [0.0537]
BWA	5380	99.78	98.17 [0.0983]	98.06 [0.0876]
BWA MEM	649	99.99	98.43 [0.0137]	98.39 [0.0083]
Bowtie2	2348	97.13	97.12 [0.9904]	94.14 [0.0083]
SOAP2	856	75.25	74.04 [0.3487]	
SeqAlto	7191	99.58	97.80 [0.0723]	97.74 [0.0698]

Time measurement is elapsed time (second). Unique refers to MAPQ ≥ 1 if MAPQ available. Q10 refers to MAPQ ≥ 10

#### Results of real datasets

We ran six of the aligners with various parameter settings for one paired-end reads and two single-end reads. All results can be found in the Additional file 1.

Table 4 shows the best results from the total number of reads aligned for single-end reads and paired-end reads. For two single-ends, HIA and BWA MEM are higher ranks than the other aligners in terms of the speed and the total number of reads aligned. For the paired-end reads, the aligned percentage of Bowtie2 is higher than the other aligners, but HIA is faster than all of the other aligners except SOAP2.

**Table 4:** Results for real datasets

Aligner	Time	% Aligned	% Unique	% Q10
(a) Illumina 100 bp reads (unpaired)				
HIA	369	97.71	91.23	86.41
BWA	2877	85.87	81.82	81.68
BWA MEM	272	96.86	89.32	86.85
Bowtie2	1291	94.96	92.11	83.69
SOAP2	283	87.29	82.29	
SeqAlto	1567	89.16	85.22	84.60
(b) 454 400 bp reads (unpaired)				
HIA	964	99.05	96.90	95.92
BWA-SW	6369	99.53	96.48	92.63
BWA MEM	830	99.73	96.36	94.86
Bowtie2	6597	98.37	96.96	91.02
(c) Illumina 100 bp reads (paired)				
HIA	1111	91.53	87.48	85.25
BWA	2871	88.90	86.59	86.26
BWA MEM	690	93.49	90.55	89.80
Bowtie2	1646	93.13	91.60	84.93
SOAP2	725	82.18	79.56	
SeqAlto	3370	92.04	87.82	87.55

Time measurement is elapsed time (second). Unique refers to MAPQ ≥ 1 if MAPQ available. Q10 refers to MAPQ ≥ 10

#### Results of multithreading tests

We ran six of the aligners with 6 threads and 12 threads modes for the whole of the paired-end HiSeq reads. All results can be found in the Table 5. HIA is faster than the other aligners for both 6 threads and 12 threads modes.

**Table 5:** Results of the multithreading tests

Aligner	Time (6 threads)	Time (12 threads)
HIA	932	505
BWA	4006	2586
BWA MEM	1162	645
bowtie2	1180	789
soap2	2217	1616
seqAlto	3945	2077

Time measurement is elapsed time (minute)

## Conclusions

We developed a new sequence alignment tool for aligning short and long reads to a reference genome. HIA has two indexes, a HT index and a SA index. The HT is capable of direct lookup of a q-gram, and the SA can very rapidly look up q-grams of variable length. Our experiments show that the hybrid of HT and SA is useful, from the perspective of speed, for mapping NGS sequencing reads to a reference genome sequence. HIA also supports the multithreading of mapping. In particular, HIA is much faster than all of the other aligners; therefore, our tool is appropriate for aligning massive data sets generated by NGS sequencing.

The accuracy of alignment is very important in re-sequencing because the main purpose of alignment is to discover the variants relative to a reference genome. Although these variants (or sequencing errors) cause sequencing reads within them to match inexactly to the reference, alignment tools should nonetheless correctly map these reads to the reference. Considering the results of the benchmark analysis, we can conclude that HIA performs comparably to four popular alignment tools.

## Availability and requirements

- Project name: HIA
- Project home page: http://biomi.cdc.go.kr/hia/
- Operating systems: Platform independent
- Programming language
- License: GNU GPL
- Any restrictions to use by non-academics: none.

## Additional files

10.1186/s13015-015-0062-4 It includes the test datasets, all tested results, and information of all used tools.
10.1186/s13015-015-0062-4 It contains the jar file of HIA. HIA can be used without restriction.

## Abbreviations

HIA: hybrid index based sequence alignment
GS: next-generation sequencing
CAR: candidate alignment region
MR: matching regions
HT: hash table
BWT: Burrows–Wheeler transform
SA: suffix array
IDF: inverse document frequency
LMUR: leftmost unmatched region
RMUR: rightmost unmatched region
MRUR: unmatched region between two MRs
FASTA: FAST-All
SAM: sequence alignment/map format
GRCh37: genome reference consortium human genome build 37
MAPQ: mapping quality
