# Supplementary material for: HIA: a genome mapper using hybrid index-based sequence alignment
Source: Algorithms Mol Biol. 2015 Dec 23;10:30. doi: 10.1186/s13015-015-0062-4 (PMC4688996; doi:10.1186/s13015-015-0062-4)
Supplement: Supplementary file 2 — 10.1186/s13015-015-0062-4 It contains the jar file of HIA. HIA can be used without restriction. [file 13015_2015_62_MOESM2_ESM.pdf]

HIA: A genome mapper using Hybrid Index-based sequence Alignment

Jongpill Choi, Kiejung Park, Seong Beom Cho and Myungguen Chung

1. Versions

This lists the version numbers of each aligners used in the evaluation.

	Aligner	Version
1	HIA	hia-1.0
2	BWA	bwa-0.6.1, bwa-0.7.7
3	Bowtie2	bowtie2-2.1.0
4	Soap2	soap2.21realease
5	SeqAlto	0.5-r123_x86_64(seqalto_basic)

2. Summary of the parameters used in tests

All aligners supply many parameters to tune the aligners in various situations. It is hard to test all possible parameter settings so that we select some parameter settings and test the aligners for the simulated datasets and the real datasets. We summarize the parameters used in the tests as follows:

Aligner	Options	Description
HIA	-r FLOAT	the error rate of reads; by default, 0.021
	-x INT	the max frequency of q-grams; by default, 100
	-s INT	the step size; by default, 4
BWA [aln]	-k INT	maximum differences in the seed [2]
	-l INT	seed length [32]
	-o INT	maximum number or fraction of gap opens [1]
BWA [bwasw]	-c FLOAT	coefficient of length-threshold adjustment [5.5]
	-z INT	Z-best [1]
	-s INT	maximum seeding interval size [3]
BWA [mem]	-k INT	minimum seed length [19]
	-r FLOAT	look for internal seeds inside a seed longer than {-k} * FLOAT [1.5]
Bowtie2	-D INT	give up extending after <int> failed extends in a row (15)
	-R INT	for reads w/ repetitive seeds, try <int> sets of seeds (2)
	-L INT	length of seed substrings; must be >3, <32 (22)
	-i <func>	interval between seed substrings w/r/t read len (S,1,1.25)
SOAP2	-l INT	align the initial n bps as a seed [256] means whole length of read
	-v INT	maximum number of mismatches allowed on a read. [5] bp
SeqAlto	-f	Activates fast alignment mode, default off
	-a	Activates accurate alignment mode, default off
	-u	Disables gapped alignment, default off

3. Simulated Reads

Six datasets were simulated from the GRCH37 build of the human genome using the Mason simulator (http://www.seqan.de/projects/mason.html). The following are the command line parameters to simulate each dataset.

Illumina-like 100 bp (unpaired) dataset

Usage: mason illumina [Options] SEQUENCE
Options: -hn 2 -sq -n 100 -N 1000000 -rnp ill_100 –o output-file
SEQUENCE: hg19.fa (including sex chromosomes and mitochondrial genome)
Description of given options
- -hn: Number of haplotypes to simulate
- -sq: Simulate qualities, generate FASTQ instead of FASTA
- -n: The length of the reads to simulate (All resulting reads are the same length)
- -N: Number of reads to simulate
- -rnp: Read name prefix
- -o: output file name to write results

Illumina-like 150 bp (unpaired) dataset

Usage: mason illumina [Options] SEQUENCE
Options: -hn 2 -sq -n 150 -N 1000000 -rnp ill_150 –o output-file
SEQUENCE: hg19.fa (including sex chromosomes and mitochondrial genome)
Description of given options
- -hn: Number of haplotypes to simulate
- -sq: Simulate qualities, generate FASTQ instead of FASTA
- -n: The length of the reads to simulate (All resulting reads are the same length)
- -N: Number of reads to simulate
- -rnp: Read name prefix
- -o: output file name to write results

Illumina-like 100 bp (paired-end) dataset

Usage: mason illumina [Options] SEQUENCE
Options: -hn 2 -sq -rn 2 -mp -ll 375 -le 100 -n 100 -N 1000000 -rnp ill_100_pe -o output-file
SEQUENCE: hg19.fa (including sex chromosomes and mitochondrial genome)
Description of given options
- -hn: Number of haplotypes to simulate
- -sq: Simulate qualities, generate FASTQ instead of FASTA
- -rn: Read name scheme in FASTQ files, 0-2
  - 2 = Add one-based slash-suffix, i.e. names will end on '/1' and '/2'
- -mp: Enable mate pair simulation
- -ll: Mate-pair mean library length
- -le: Mate-pair library tolerance
- -n: The length of the reads to simulate (All resulting reads are the same length)
- -N: Number of reads to simulate
- -rnp: Read name prefix
- -o: output file name to write results

Illumina-like 150 bp (paired-end) dataset

Usage: mason illumina [Options] SEQUENCE
Options: -hn 2 -sq -rn 2 -mp -ll 375 -le 100 -n 150 -N 1000000 -rnp ill_100_pe -o output-file
SEQUENCE: hg19.fa (including sex chromosomes and mitochondrial genome)
Description of given options
- -hn: Number of haplotypes to simulate
- -sq: Simulate qualities, generate FASTQ instead of FASTA
- -rn: Read name scheme in FASTQ files, 0-2
  - 2 = Add one-based slash-suffix, i.e. names will end on '/1' and '/2'
- -mp: Enable mate pair simulation
- -ll: Mate-pair mean library length
- -le: Mate-pair library tolerance
- -n: The length of the reads to simulate (All resulting reads are the same length)

-N: Number of reads to simulate
-rnp: Read name prefix
-o: output file name to write results

454-like 250 bp (unpaired) dataset
Usage: mason 454 [Options] SEQUENCE
Options: -hn 2 -sq -k 0.3 -bm 0.4 -bs 0.2 -nm 250 -N 1000000 -rnp 454_250 –o output-file
SEQUENCE: hg19.fa (including sex chromosomes and mitochondrial genome)
Description of given options
-hn: Number of haplotypes to simulate
-sq: Simulate qualities, generate FASTQ instead of FASTA
-k: Proportionality factor for calculating standard deviation proportional to sqrt(homoplymer length)
-bm: Background noise mean
-bs: Background noise standard deviation
-nm: The mean of the read lengths
-N: Number of reads to simulate
-rnp: Read name prefix
-o: output file name to write results

454-like 400 bp (unpaired) dataset
Usage: mason 454 [Options] SEQUENCE
Options: -hn 2 -sq -k 0.3 -bm 0.4 -bs 0.2 -nm 250 -N 1000000 -rnp 454_250 –o output-file
SEQUENCE: hg19.fa (including sex chromosomes and mitochondrial genome)
Description of given options
-hn: Number of haplotypes to simulate
-sq: Simulate qualities, generate FASTQ instead of FASTA
-k: Proportionality factor for calculating standard deviation proportional to sqrt(homoplymer length)
-bm: Background noise mean
-bs: Background noise standard deviation
-nm: The mean of the read lengths
-N: Number of reads to simulate
-rnp: Read name prefix
-o: output file name to write results

4. Real Reads

The real Illumina datasets are based on the 101 bp×99 bp paired-end HiSeq (2000) reads from a human re-sequencing study [20]. It consists of 1,296,188,286 reads (101 bp×99 bp paired-end). The 454 dataset has NCBI Short Read Archive accession number SRR003161 and contains 1,375,489 reads with an average length of 355bp. For the performance tests, we prepared four datasets as follows:

Whole of HiSeq 101bp×99 bp (paired-end) dataset
NCBI Short Read Archive accession number SRR341919
It contains 1,296,188,286 (648094143×2) reads for the multi-thread test.

HiSeq 101 bp (single-end) dataset
It contains one million 101 bp reads of the HiSeq reads for single-end reads.

HiSeq 101bp×99 bp (paired-end) dataset
It contains one million 101 bp×99 bp paired-end reads of the HiSeq reads for paired-end reads.

454-like 400 bp (single-end) dataset
NCBI Short Read Archive accession number SRR003161
It contains 1,375,489 reads with an average length of 355bp.

5. Results for index generation

The following is the command-line parameters and the results for indexing the GRCH37 build of the human genome. We download the GRCH37 build of the human genome (hg19) from UCSC Genome browser site. The reference genome used for our experiments consists of 25 chromosomes and its size is about 2.9 GB. The

indexing time of HIA is comparable to other aligners. Especially, HIA is able to reduce the time of index generation by using multiple threads.

Aligner	Options	Time (M)	Memory	Size	Generated files
HIA	-t 1 -q 14	165	20.32 GB	12.63 GB	*.SA, *.idx, *.seq, and *.seqinfo
HIA	-t 12 -q 14	28	20.47 GB	12.63 GB	*.SA, *.idx, *.seq, and *.seqinfo
BWA		69	4.53 GB	5.40GB	*.amb, *.ann, *.bwt, *.pac, and *.sa
bowtie2		99	5.35 GB	4.10 GB	*.1.bt2, *.2.bt2, *.3.bt2, *.4.bt2, *.rev.1.bt2, and *.rev.2.bt2
soap2		55	3.39 GB	5.90 GB	*.amb, *.ann, *.bwt, *.fmv, *.hot, *.lkt, *.pac, *.rev.bwt, *.rev.fmv, *.rev.lkt, *.rev.pac, *.sa, and *.sai
seqAlto	-I 0 genome.fa 28	33	37.99 GB	22.40 GB	*.midx
seqAlto	-I 1 genome.fa 22	12	13.19 GB	5.52 GB	*.sidx

Time measurement is elapsed time (minute). Memory is the peak memory for the index construction. Size is the sum of all generated files.

In the perspective of SA construction, we performed several tests of index generation and compared the results to the result of divsufsort. Divsufsort algorithm is faster than HIA. However, HIA can reduce the generation time using multithreads and applying various q-gram sizes.

**Aligner**	**Options**	**Time (M)**
HIA	-t 1 -q 14	165
HIA	-t 12 -q 2	23
HIA	-t 12 -q 4	18
HIA	-t 12 -q 6	17
HIA	-t 12 -q 8	17
HIA	-t 12 -q 10	18
HIA	-t 12 -q 12	21
HIA	-t 12 -q 14	28
divsufsort		13

Time measurement is elapsed time (minute).

6. Performance of HT plus SA and the unmatched region alignment approach

We performed some tests using the simulated dataset (Illumina-like 100 bp [paired-end]) to observe the benefit of HT plus SA and the benefit of aligning the unmatched regions. The following result shows that the proposed approach can reduce overall alignment time.

	**Time (S)**
Proposed approach	497
Aligning CAR by only NW	907
Finding CARs by only SA	1060

Time measurement is elapsed time (second).

7. Memory consumption tests

We performed some tests using the simulated dataset (Illumina-like 100 bp [paired-end]) to observe the memory consumption of six aligners. We use the memusg shell script (https://gist.github.com/netj/526585) to measure the peak memory of each tool. HIA needs more memory than BWA, bowtie2, and soap2.

Aligner	Options	Memory
HIA	-s 4 -r 0.021 -x 200	18.73 GB
BWA	-k 2 -l 32 -o 1	3.28 GB
BWA MEM	-k 19 -r 1.5	5.40 GB
bowtie2	-D 15 -R 2 -N 0 -L 22 -i S,1,1.5	3.29 GB
soap2	-l 40 -v 3 -g 3 -m 250 -x 500	5.56 GB
seqAlto	hg19.midx -a	23.68 GB

Memory is the peak memory.

8. Results of the multithreading tests

We ran six of the aligners with 6 threads and 12 threads modes for the whole of the paired-end HiSeq reads. HIA is faster than the other aligners for both 6 threads and 12 threads modes.

Aligner	Options	Time (6 threads)	Time (12 threads)
HIA	-s 4 -r 0.021 -x 200	932	505
BWA	-k 2 -l 32 -o 1	4006	2586
BWA MEM	-k 19 -r 1.5	1162	645
bowtie2	-D 15 -R 2 -N 0 -L 22 -i S,1,1.5	1180	789
soap2	-l 40 -v 3 -g 3 -m 250 -x 500	2217	1616
seqAlto	hg19.midx -a	3945	2077

Time measurement is elapsed time (minute).

9. Results for simulated datasets

The following are the results for simulated datasets. Time measure is elapsed time (second). Unique refers to MAPQ ≥ 1 if MAPQ is reported. Q10 refers to MAPQ ≥ 10. %Err refers to the percentage aligned incorrectly among the reads in each criteria.

For both Illumina-like datasets and 454-like datasets, HIA is significantly faster than all of the other aligners except BWA MEM and SOAP2. SOAP2 is very fast, but not as sensitive as HIA. BWA is slightly more accurate, but not as sensitive as HIA for Illumina-like datasets. HIA is more sensitive and accurate than BWA for 454-like datasets. Bowtie2 is similar to HIA with regard to sensitivity, but not as accurate as HIA for both Illumina-like datasets and 454-like datasets. SeaAlto SeqAlto is slightly more accurate, but not as sensitive as HIA for Illumina-like datasets. BWA MEM is more accurate and more sensitive than HIA for Illumina-like datasets, but not as sensitive as HIA for 454-like datasets.

Illumina-like 100 bp (single-end) dataset

	Aligner	Options	Time	%Aligned	%Unique	[%Err]		%Q10	[%Err]
1	HIA	-r 0.011 -x 50 -s 4	217	99.95	96.63	[0.4079]		94.74	[0.1674]
2	HIA	-r 0.011 -x 100 -s 4	221	99.97	96.62	[0.3607]		94.69	[0.1450]
3	HIA	-r 0.011 -x 200 -s 4	249	99.98	96.62	[0.3164]		94.66	[0.1273]
4	HIA	-r 0.011 -x 300 -s 4	264	99.98	96.62	[0.2971]		94.65	[0.1181]
5	HIA	-r 0.021 -x 50 -s 4	195	100.00	96.64	[0.5340]		95.36	[0.2406]
6	HIA	-r 0.021 -x 100 -s 4	237	100.00	96.63	[0.4886]		95.36	[0.1952]
7	HIA	-r 0.021 -x 200 -s 4	262	100.00	96.61	[0.3654]		95.35	[0.1661]
8	HIA	-r 0.021 -x 300 -s 4	307	100.00	96.60	[0.3267]		95.35	[0.1510]
9	HIA	-r 0.031 -x 50 -s 4	249	100.00	96.60	[0.8353]		95.42	[0.3669]
10	HIA	-r 0.031 -x 100 -s 4	312	100.00	96.58	[0.6725]		95.43	[0.2884]
11	HIA	-r 0.031 -x 200 -s 4	368	100.00	96.58	[0.5300]		95.45	[0.2272]
12	HIA	-r 0.031 -x 300 -s 4	399	100.00	96.57	[0.4610]		95.46	[0.2026]
13	HIA	-r 0.041 -x 50 -s 4	277	100.00	96.60	[0.7832]		95.74	[0.3823]
14	HIA	-r 0.041 -x 100 -s 4	328	100.00	96.58	[0.6321]		95.75	[0.3018]
15	HIA	-r 0.041 -x 200 -s 4	432	100.00	96.58	[0.4983]		95.78	[0.2413]
16	HIA	-r 0.041 -x 300 -s 4	464	100.00	96.57	[0.4314]		95.80	[0.2151]

	Aligner	Options	Time	%Aligned	%Unique	[%Err]		%Q10	[%Err]
1	BWA	-k 1 -l 32 -o 1	207	93.27	90.03	[0.1812]		89.91	[0.1678]
2	BWA	-k 1 -l 32 -o 2	236	94.70	91.43	[0.1795]		91.31	[0.1660]
3	BWA	-k 1 -l 28 -o 1	260	93.82	90.57	[0.1834]		90.44	[0.1702]

4	BWA	-k 1 -l 32 -o 3	245	94.78	91.50	[0.1799]		91.38	[0.1663]
5	BWA	-k 1 -l 28 -o 2	304	95.34	92.04	[0.1818]		91.92	[0.1684]
6	BWA	-k 1 -l 24 -o 1	347	94.40	91.13	[0.1849]		91.00	[0.1716]
7	BWA	-k 1 -l 28 -o 3	320	95.42	92.12	[0.1818]		92.00	[0.1684]
8	BWA	-k 1 -l 24 -o 2	410	95.98	92.67	[0.1825]		92.54	[0.1691]
9	BWA	-k 1 -l 24 -o 3	471	96.07	92.75	[0.1824]		92.63	[0.1690]
10	BWA	-k 2 -l 32 -o 1	658	96.01	92.69	[0.1738]		92.56	[0.1596]
11	BWA	-k 2 -l 28 -o 1	855	96.19	92.86	[0.1740]		92.73	[0.1596]
12	BWA	-k 2 -l 32 -o 2	843	97.70	94.34	[0.1713]		94.21	[0.1568]
13	BWA	-k 2 -l 32 -o 3	758	97.81	94.44	[0.1714]		94.31	[0.1569]
14	BWA	-k 2 -l 28 -o 2	960	97.90	94.54	[0.1711]		94.41	[0.1566]
15	BWA	-k 2 -l 24 -o 1	1111	96.37	93.04	[0.1742]		92.91	[0.1595]
16	BWA	-k 2 -l 28 -o 3	980	98.02	94.64	[0.1713]		94.51	[0.1566]
17	BWA	-k 2 -l 24 -o 2	1242	98.11	94.73	[0.1711]		94.60	[0.1562]
18	BWA	-k 2 -l 24 -o 3	1266	98.21	94.83	[0.1711]		94.70	[0.1563]

	Aligner	Options	Time	%Aligned	%Unique	[%Err]		%Q10	[%Err]
1	BWA MEM	-k 17 -r 1.3	265	100.00	96.30	[0.0497]		95.27	[0.0153]
2	BWA MEM	-k 17 -r 1.5	265	100.00	96.30	[0.0490]		95.27	[0.0155]
3	BWA MEM	-k 17 -r 1.7	255	100.00	96.30	[0.0494]		95.27	[0.0155]
4	BWA MEM	-k 19 -r 1.3	240	100.00	96.30	[0.0502]		95.27	[0.0158]
5	BWA MEM	-k 19 -r 1.5	244	100.00	96.30	[0.0504]		95.27	[0.0157]
6	BWA MEM	-k 19 -r 1.7	234	100.00	96.30	[0.0514]		95.27	[0.0158]
7	BWA MEM	-k 21 -r 1.3	241	100.00	96.30	[0.0515]		95.27	[0.0162]
8	BWA MEM	-k 21 -r 1.5	232	100.00	96.30	[0.0523]		95.27	[0.0163]
9	BWA MEM	-k 21 -r 1.7	234	100.00	96.30	[0.0534]		95.27	[0.0172]

	Aligner	Options	Time	%Aligned	%Unique	[%Err]		%Q10	[%Err]
1	Bowtie2	-D 5 -R 1 -N 0 -L 22 -i S,0,2.50	220	98.97	98.42	[2.6245]		93.25	[0.0646]
2	Bowtie2	-D 10 -R 2 -N 0 -L 22 -i S,0,2.50	306	99.52	99.03	[2.6145]		93.70	[0.0392]
3	Bowtie2	-D 15 -R 2 -N 0 -L 22 -i S,1,2.50	346	99.63	99.17	[2.5870]		93.81	[0.0336]
4	Bowtie2	-D 15 -R 2 -N 0 -L 22 -i S,1,2.20	346	99.60	99.12	[2.5941]		93.76	[0.0354]

5	Bowtie2	-D 15 -R 2 -N 0 -L 22 -i S,1,1.65	386	99.70	99.28	[2.5423]		93.92	[0.0328]
6	Bowtie2	-D 15 -R 2 -N 0 -L 22 -i S,1,1.15	439	99.83	99.44	[2.5085]		94.08	[0.0293]
7	Bowtie2	-D 15 -R 2 -N 0 -L 21 -i S,1,1.00	497	99.84	99.44	[2.5273]		94.06	[0.0298]
8	Bowtie2	-D 15 -R 2 -N 0 -L 20 -i S,1,0.75	636	99.85	99.45	[2.5100]		94.08	[0.0291]
9	Bowtie2	-D 15 -R 2 -N 0 -L 20 -i S,1,0.50	727	99.85	99.46	[2.4978]		94.09	[0.0291]
10	Bowtie2	-D 20 -R 3 -N 0 -L 20 -i S,1,0.50	999	99.93	99.59	[2.4551]		94.19	[0.0237]
11	Bowtie2	-D 25 -R 4 -N 0 -L 20 -i S,1,0.50	1291	99.95	99.63	[2.4252]		94.22	[0.0208]

	Aligner	Options	Time	%Aligned	%Unique	[%Err]		%Q10	[%Err]
1	SOAP2	-l 256 -v 3 -g 0	149	76.19	73.56	[0.1055]		73.56	[0.1055]
2	SOAP2	-l 256 -v 5 -g 0	154	76.19	73.56	[0.1055]		73.56	[0.1055]
3	SOAP2	-l 256 -v 7 -g 0	145	76.19	73.56	[0.1055]		73.56	[0.1055]
4	SOAP2	-l 75 -v 5 -g 0	195	77.98	75.23	[0.2071]		75.23	[0.2071]
5	SOAP2	-l 75 -v 7 -g 0	194	78.61	75.83	[0.2117]		75.83	[0.2117]
6	SOAP2	-l 75 -v 3 -g 0	196	77.32	74.61	[0.1921]		74.61	[0.1921]
7	SOAP2	-l 40 -v 7 -g 0	264	79.37	76.27	[0.4679]		76.27	[0.4679]
8	SOAP2	-l 40 -v 5 -g 0	294	78.52	75.60	[0.3772]		75.60	[0.3772]
9	SOAP2	-l 40 -v 3 -g 0	319	77.65	74.89	[0.2572]		74.89	[0.2572]
7	SOAP2	-l 28-v 7 -g 0		0.00		[0.0000]			[0.0000]
8	SOAP2	-l 28 -v 5 -g 0		0.00		[0.0000]			[0.0000]
9	SOAP2	-l 28 -v 3 -g 0		0.00		[0.0000]			[0.0000]

	Aligner	Options	Time	%Aligned	%Unique	[%Err]		%Q10	[%Err]
1	SeqAlto	-f [Index mode: 0, k-mer_size: 28]	688	99.38	96.38	[0.3795]		96.11	[0.2934]
2	SeqAlto	-a [Index mode: 0, k-mer_size: 28]	1513	99.59	96.30	[0.2901]		96.00	[0.2240]
3	SeqAlto	-f -u [Index mode: 0, k-mer_size: 28]	396	80.22	77.73	[0.3831]		77.53	[0.3016]
4	SeqAlto	-a -u [Index mode: 0, k-mer_size: 28]	466	80.57	77.77	[0.3333]		77.51	[0.2583]
5	SeqAlto	-f [Index mode: 1, k-mer_size: 22]	451	99.21	96.22	[0.4684]		95.94	[0.3492]
6	SeqAlto	-a [Index mode: 1, k-mer_size: 22]	1459	99.69	96.33	[0.2861]		96.04	[0.2156]
7	SeqAlto	-f -u [Index mode: 1, k-mer_size: 22]	127	80.07	77.60	[0.4437]		77.40	[0.3355]
8	SeqAlto	-a -u [Index mode: 1, k-mer_size: 22]	217	80.64	77.78	[0.3314]		77.53	[0.2541]

Illumina-like 150 bp (single-end) dataset

	Aligner	Options	Time	%Aligned	%Unique [%Err]		%Q10 [%Err]
1	HIA	-r 0.011 -x 50 -s 4	221	99.98	97.72 [0.2600]		96.08 [0.0861]
2	HIA	-r 0.011 -x 100 -s 4	250	99.99	97.62 [0.2381]		96.06 [0.0777]
3	HIA	-r 0.011 -x 200 -s 4	274	99.99	97.61 [0.2175]		96.03 [0.0691]
4	HIA	-r 0.011 -x 300 -s 4	275	99.99	97.60 [0.2025]		96.01 [0.0641]
5	HIA	-r 0.021 -x 50 -s 4	255	100.00	97.59 [0.2758]		96.77 [0.1515]
6	HIA	-r 0.021 -x 100 -s 4	301	100.00	97.58 [0.2310]		96.73 [0.1266]
7	HIA	-r 0.021 -x 200 -s 4	350	100.00	97.57 [0.1931]		96.69 [0.1069]
8	HIA	-r 0.021 -x 300 -s 4	369	100.00	97.56 [0.1781]		96.67 [0.0988]
9	HIA	-r 0.031 -x 50 -s 4	301	100.00	97.58 [0.3525]		96.96 [0.2183]
10	HIA	-r 0.031 -x 100 -s 4	372	100.00	97.57 [0.2854]		96.91 [0.1703]
11	HIA	-r 0.031 -x 200 -s 4	420	100.00	97.57 [0.2318]		96.86 [0.1380]
12	HIA	-r 0.031 -x 300 -s 4	510	100.00	97.56 [0.2024]		96.83 [0.1213]
13	HIA	-r 0.041 -x 50 -s 4	363	100.00	97.58 [0.4528]		97.27 [0.3092]
14	HIA	-r 0.041 -x 100 -s 4	378	100.00	97.58 [0.3793]		97.26 [0.2420]
15	HIA	-r 0.041 -x 200 -s 4	494	100.00	97.57 [0.3012]		97.26 [0.1936]
16	HIA	-r 0.041 -x 300 -s 4	530	100.00	97.56 [0.2552]		97.26 [0.1643]

	Aligner	Options	Time	%Aligned	%Unique [%Err]		%Q10 [%Err]
1	BWA	-k 1 -l 32 -o 1	417	90.17	87.89 [0.1105]		87.82 [0.1034]
2	BWA	-k 1 -l 32 -o 2	541	93.78	91.41 [0.1047]		91.34 [0.0973]
3	BWA	-k 1 -l 28 -o 1	541	90.84	88.54 [0.1118]		88.47 [0.1044]
4	BWA	-k 1 -l 32 -o 3	644	94.10	91.72 [0.1038]		91.65 [0.0965]
5	BWA	-k 1 -l 28 -o 2	698	94.54	92.16 [0.1049]		92.09 [0.0973]
6	BWA	-k 1 -l 24 -o 1	730	91.50	89.18 [0.1102]		89.12 [0.1024]
7	BWA	-k 1 -l 28 -o 3	760	94.87	92.39 [0.1040]		92.33 [0.0964]
8	BWA	-k 1 -l 24 -o 2	929	95.31	92.91 [0.1035]		92.84 [0.0955]
9	BWA	-k 1 -l 24 -o 3	1006	95.65	93.24 [0.1024]		93.18 [0.0944]
10	BWA	-k 2 -l 32 -o 1	1214	93.18	90.83 [0.1094]		90.76 [0.0956]
11	BWA	-k 2 -l 28 -o 1	1581	93.39	91.03 [0.1045]		90.96 [0.0961]

12	BWA	-k 2 -l 32 -o 2	1449	97.17	94.73	[0.0967]		94.66	[0.0881]
13	BWA	-k 2 -l 32 -o 3	1513	97.53	95.09	[0.0956]		95.02	[0.0870]
14	BWA	-k 2 -l 28 -o 2	1879	97.40	94.96	[0.0968]		94.89	[0.0881]
15	BWA	-k 2 -l 24 -o 1	2110	93.58	91.23	[0.1048]		91.16	[0.0963]
16	BWA	-k 2 -l 28 -o 3	1945	97.78	95.33	[0.0957]		95.26	[0.0870]
17	BWA	-k 2 -l 24 -o 2	2770	97.63	95.18	[0.0964]		95.02	[0.0878]
18	BWA	-k 2 -l 24 -o 3	2464	98.00	95.55	[0.0953]		95.48	[0.0866]

	Aligner	Options	Time	%Aligned	%Unique	[%Err]		%Q10	[%Err]
1	BWA MEM	-k 17 -r 1.3	355	100.00	97.36	[0.0210]		96.41	[0.0053]
2	BWA MEM	-k 17 -r 1.5	343	100.00	97.36	[0.0213]		96.41	[0.0054]
3	BWA MEM	-k 17 -r 1.7	339	100.00	97.36	[0.0217]		96.41	[0.0057]
4	BWA MEM	-k 19 -r 1.3	338	100.00	97.36	[0.0214]		96.41	[0.0055]
5	BWA MEM	-k 19 -r 1.5	308	100.00	97.36	[0.0216]		96.41	[0.0057]
6	BWA MEM	-k 19 -r 1.7	310	100.00	97.36	[0.0219]		96.41	[0.0058]
7	BWA MEM	-k 21 -r 1.3	298	100.00	97.36	[0.0215]		96.41	[0.0058]
8	BWA MEM	-k 21 -r 1.5	299	100.00	97.36	[0.0219]		96.41	[0.0059]
9	BWA MEM	-k 21 -r 1.7	292	100.00	97.36	[0.0223]		96.41	[0.0059]

	Aligner	Options	Time	%Aligned	%Unique	[%Err]		%Q10	[%Err]
1	Bowtie2	-D 5 -R 1 -N 0 -L 22 -i S,0,2.50	307	98.89	98.53	[1.9995]		94.68	[0.0653]
2	Bowtie2	-D 10 -R 2 -N 0 -L 22 -i S,0,2.50	436	99.59	99.34	[1.8955]		95.42	[0.0289]
3	Bowtie2	-D 15 -R 2 -N 0 -L 22 -i S,1,2.50	528	99.69	99.47	[1.8662]		95.55	[0.0222]
4	Bowtie2	-D 15 -R 2 -N 0 -L 22 -i S,1,2.20	537	99.71	99.50	[1.8489]		95.58	[0.0212]
5	Bowtie2	-D 15 -R 2 -N 0 -L 22 -i S,1,1.65	580	99.84	99.66	[1.7854]		95.78	[0.0194]
6	Bowtie2	-D 15 -R 2 -N 0 -L 22 -i S,1,1.15	670	99.88	99.72	[1.7621]		95.84	[0.0192]
7	Bowtie2	-D 15 -R 2 -N 0 -L 21 -i S,1,1.00	750	99.86	99.68	[1.7650]		95.81	[0.0207]
8	Bowtie2	-D 15 -R 2 -N 0 -L 20 -i S,1,0.75	947	99.87	99.71	[1.7596]		95.85	[0.0202]
9	Bowtie2	-D 15 -R 2 -N 0 -L 20 -i S,1,0.50	1122	99.88	99.73	[1.7493]		95.86	[0.0192]
10	Bowtie2	-D 20 -R 3 -N 0 -L 20 -i S,1,0.50	1562	99.95	99.82	[1.7017]		95.95	[0.0133]
11	Bowtie2	-D 25 -R 4 -N 0 -L 20 -i S,1,0.50	2069	99.97	99.87	[1.6663]		95.99	[0.0094]

	Aligner	Options	Time	%Aligned	%Unique	[%Err]		%Q10	[%Err]
1	SOAP2	-l 256 -v 3 -g 0	213	64.61	63.00	[0.0560]		63.00	[0.0560]
2	SOAP2	-l 256 -v 5 -g 0	213	64.61	63.00	[0.0560]		63.00	[0.0560]
3	SOAP2	-l 256 -v 7 -g 0	212	64.61	63.00	[0.0560]		63.00	[0.0560]
4	SOAP2	-l 75 -v 5 -g 0	301	67.63	65.86	[0.1742]		65.86	[0.1742]
5	SOAP2	-l 75 -v 7 -g 0	297	68.25	66.42	[0.1999]		66.42	[0.1999]
6	SOAP2	-l 75 -v 3 -g 0	306	66.95	65.24	[0.1346]		65.24	[0.1346]
7	SOAP2	-l 40 -v 7 -g 0	525	68.72	66.78	[0.2806]		66.78	[0.2806]
8	SOAP2	-l 40 -v 5 -g 0	549	68.01	66.19	[0.2200]		66.19	[0.2200]
9	SOAP2	-l 40 -v 3 -g 0	557	67.19	65.48	[0.1378]		65.48	[0.1378]
10	SOAP2	-l 28-v 7 -g 0		0.00		[0.0000]			[0.0000]
11	SOAP2	-l 28 -v 5 -g 0		0.00		[0.0000]			[0.0000]
12	SOAP2	-l 28 -v 3 -g 0		0.00		[0.0000]			[0.0000]

	Aligner	Options	Time	%Aligned	%Unique	[%Err]		%Q10	[%Err]
1	SeqAlto	-f [Index mode: 0, k-mer_size: 28]	1178	99.61	97.34	[0.2554]		97.20	[0.2057]
2	SeqAlto	-a [Index mode: 0, k-mer_size: 28]	3586	99.65	97.26	[0.2035]		97.09	[0.1563]
3	SeqAlto	-f -u [Index mode: 0, k-mer_size: 28]	398	71.59	69.95	[0.2984]		69.84	[0.2421]
4	SeqAlto	-a -u [Index mode: 0, k-mer_size: 28]	491	71.81	70.00	[0.2626]		69.86	[0.1977]
5	SeqAlto	-f [Index mode: 1, k-mer_size: 22]	976	99.54	97.27	[0.2752]		97.14	[0.2199]
6	SeqAlto	-a [Index mode: 1, k-mer_size: 22]	3608	99.68	97.25	[0.1947]		97.10	[0.1490]
7	SeqAlto	-f -u [Index mode: 1, k-mer_size: 22]	142	71.56	69.91	[0.3093]		69.81	[0.2490]
8	SeqAlto	-a -u [Index mode: 1, k-mer_size: 22]	247	71.85	70.01	[0.2470]		69.88	[0.1873]

Illumina-like 100 bp (paired-end) dataset

	Aligner	Options	Time	%Aligned	%Unique	[%Err]		%Q10	[%Err]
1	HIA	-r 0.011 -x 50 -s 4	438	99.80	97.07	[0.1459]		96.27	[0.0644]
2	HIA	-r 0.011 -x 100 -s 4	456	99.83	97.03	[0.1419]		96.28	[0.0635]
3	HIA	-r 0.011 -x 200 -s 4	504	99.84	97.05	[0.1375]		96.28	[0.0633]
4	HIA	-r 0.011 -x 300 -s 4	551	99.85	97.03	[0.1352]		96.28	[0.0628]
5	HIA	-r 0.021 -x 50 -s 4	455	99.94	97.19	[0.1060]		96.54	[0.0489]

6	HIA	-r 0.021 -x 100 -s 4	497	99.95	97.16	[0.0914]		96.54	[0.0458]
7	HIA	-r 0.021 -x 200 -s 4	573	99.95	97.14	[0.0871]		96.54	[0.0424]
8	HIA	-r 0.021 -x 300 -s 4	663	99.95	97.12	[0.0852]		96.54	[0.0415]
9	HIA	-r 0.031 -x 50 -s 4	641	99.94	97.21	[0.1340]		96.54	[0.0669]
10	HIA	-r 0.031 -x 100 -s 4	681	99.95	92.20	[0.1139]		96.56	[0.0566]
11	HIA	-r 0.031 -x 200 -s 4	804	99.95	97.19	[0.0961]		96.58	[0.0523]
12	HIA	-r 0.031 -x 300 -s 4	903	99.96	97.18	[0.0933]		96.58	[0.0500]
13	HIA	-r 0.041 -x 50 -s 4	741	99.94	97.20	[0.1296]		96.68	[0.0727]
14	HIA	-r 0.041 -x 100 -s 4	747	99.95	97.20	[0.1049]		96.71	[0.0600]
15	HIA	-r 0.041 -x 200 -s 4	882	99.96	97.19	[0.0901]		96.74	[0.0530]
16	HIA	-r 0.041 -x 300 -s 4	1009	99.96	97.17	[0.0859]		96.75	[0.0510]

	Aligner	Options	Time	%Aligned	%Unique	[%Err]		%Q10	[%Err]
1	BWA	-k 1 -l 32 -o 1	482	99.07	97.17	[0.1020]		96.98	[0.0767]
2	BWA	-k 1 -l 32 -o 2	558	99.33	97.38	[0.1003]		97.18	[0.0735]
3	BWA	-k 1 -l 28 -o 1	598	99.18	97.26	[0.1028]		97.06	[0.0770]
4	BWA	-k 1 -l 32 -o 3	548	99.34	97.40	[0.0998]		97.19	[0.0737]
5	BWA	-k 1 -l 28 -o 2	660	99.43	97.46	[0.0997]		97.26	[0.0732]
6	BWA	-k 1 -l 24 -o 1	755	99.27	97.33	[0.1026]		97.13	[0.0761]
7	BWA	-k 1 -l 28 -o 3	708	99.44	97.46	[0.1001]		97.27	[0.0731]
8	BWA	-k 1 -l 24 -o 2	861	99.52	97.52	[0.0990]		97.32	[0.0730]
9	BWA	-k 1 -l 24 -o 3	894	99.53	97.53	[0.0995]		97.33	[0.0724]
10	BWA	-k 2 -l 32 -o 1	1367	99.52	97.51	[0.0969]		97.31	[0.0719]
11	BWA	-k 2 -l 28 -o 1	1761	99.54	97.53	[0.0969]		97.33	[0.0722]
12	BWA	-k 2 -l 32 -o 2	1517	99.73	97.67	[0.0945]		97.46	[0.0694]
13	BWA	-k 2 -l 32 -o 3	1549	99.74	97.67	[0.0947]		97.48	[0.0695]
14	BWA	-k 2 -l 28 -o 2	1592	99.76	97.69	[0.0948]		97.48	[0.0698]
15	BWA	-k 2 -l 24 -o 1	2280	99.57	97.55	[0.0976]		97.38	[0.0723]
16	BWA	-k 2 -l 28 -o 3	1994	99.77	97.69	[0.0946]		97.48	[0.0692]
17	BWA	-k 2 -l 24 -o 2	2518	99.78	97.70	[0.0951]		97.49	[0.0699]
18	BWA	-k 2 -l 24 -o 3	2554	99.79	97.70	[0.0954]		97.49	[0.0692]

	Aligner	Options	Time	%Aligned	%Unique	[%Err]		%Q10	[%Err]
1	BWA MEM	-k 17 -r 1.3	646	99.99	98.03	[0.0282]		97.95	[0.0172]
2	BWA MEM	-k 17 -r 1.5	635	99.99	98.03	[0.0283]		97.95	[0.0173]
3	BWA MEM	-k 17 -r 1.7	623	99.99	98.03	[0.0284]		97.95	[0.0174]
4	BWA MEM	-k 19 -r 1.3	598	99.99	98.03	[0.0288]		97.95	[0.0178]
5	BWA MEM	-k 19 -r 1.5	592	99.99	98.03	[0.0287]		97.95	[0.0178]
6	BWA MEM	-k 19 -r 1.7	586	99.99	98.03	[0.0289]		97.95	[0.0182]
7	BWA MEM	-k 21 -r 1.3	587	99.99	98.03	[0.0290]		97.95	[0.0182]
8	BWA MEM	-k 21 -r 1.5	583	99.99	98.03	[0.0290]		97.95	[0.0184]
9	BWA MEM	-k 21 -r 1.7	570	99.99	98.03	[0.0289]		97.95	[0.0183]

	Aligner	Options	Time	%Aligned	%Unique	[%Err]		%Q10	[%Err]
1	Bowtie2	-D 5 -R 1 -N 0 -L 22 -i S,0,2.50	554	96.91	96.85	[1.3958]		93.52	[0.0211]
2	Bowtie2	-D 10 -R 2 -N 0 -L 22 -i S,0,2.50	558	96.95	96.89	[1.3640]		93.54	[0.0164]
3	Bowtie2	-D 15 -R 2 -N 0 -L 22 -i S,1,2.50	631	96.97	96.91	[1.3492]		93.56	[0.0143]
4	Bowtie2	-D 15 -R 2 -N 0 -L 22 -i S,1,2.20	659	96.97	96.91	[1.3427]		93.56	[0.0142]
5	Bowtie2	-D 15 -R 2 -N 0 -L 22 -i S,1,1.65	703	97.06	97.01	[1.3220]		93.65	[0.0147]
6	Bowtie2	-D 15 -R 2 -N 0 -L 22 -i S,1,1.15	781	97.09	97.04	[1.3094]		93.69	[0.0143]
7	Bowtie2	-D 15 -R 2 -N 0 -L 21 -i S,1,1.00	854	97.10	97.06	[1.3033]		93.71	[0.0144]
8	Bowtie2	-D 15 -R 2 -N 0 -L 20 -i S,1,0.75	981	97.11	97.06	[1.3047]		93.71	[0.0141]
9	Bowtie2	-D 15 -R 2 -N 0 -L 20 -i S,1,0.50	1108	97.11	97.06	[1.3077]		93.72	[0.0137]
10	Bowtie2	-D 20 -R 3 -N 0 -L 20 -i S,1,0.50	1575	97.13	97.08	[1.2807]		93.73	[0.0131]
11	Bowtie2	-D 25 -R 4 -N 0 -L 20 -i S,1,0.50	1691	97.13	97.09	[1.2711]		93.73	[0.0128]

	Aligner	Options	Time	%Aligned	%Unique	[%Err]		%Q10	[%Err]
1	SOAP2	-l 256 -v 3 -g 0 -m 250 -x 500	322	54.61	53.39	[0.0457]			[0.0000]
2	SOAP2	-l 256 -v 5 -g 0 -m 250 -x 500	362	54.61	53.39	[0.0457]			[0.0000]
3	SOAP2	-l 256 -v 7 -g 0 -m 250 -x 500	318	54.61	53.39	[0.0457]			[0.0000]
4	SOAP2	-l 75 -v 7 -g 0 -m 250 -x 500	382	57.86	56.55	[0.0697]			[0.0000]
5	SOAP2	-l 75 -v 5 -g 0 -m 250 -x 500	382	56.97	55.68	[0.0686]			[0.0000]
6	SOAP2	-l 75 -v 3 -g 0 -m 250 -x 500	381	56.08	54.81	[0.0648]			[0.0000]
7	SOAP2	-l 75 -v 7 -g 3 -m 250 -x 500	586	84.54	82.75	[0.3356]			[0.0000]

8	SOAP2	-l 75 -v 5 -g 3 -m 250 -x 500	627	84.37	82.62	[0.3257]			[0.0000]
9	SOAP2	-l 256 -v 7 -g 3 -m 250 -x 500	561	84.27	82.49	[0.3375]			[0.0000]
10	SOAP2	-l 256 -v 5 -g 3 -m 250 -x 500	559	84.23	82.49	[0.3259]			[0.0000]
11	SOAP2	-l 75 -v 3 -g 3 -m 250 -x 500	597	83.44	81.77	[0.3000]			[0.0000]
12	SOAP2	-l 256 -v 3 -g 3 -m 250 -x 500	557	82.73	81.07	[0.2962]			[0.0000]
13	SOAP2	-l 40 -v 7 -g 0 -m 250 -x 500	663	58.34	56.98	[0.1243]			[0.0000]
14	SOAP2	-l 40 -v 5 -g 0 -m 250 -x 500	668	57.40	56.08	[0.1116]			[0.0000]
15	SOAP2	-l 40 -v 3 -g 0 -m 250 -x 500	687	56.42	55.15	[0.0881]			[0.0000]
16	SOAP2	-l 40 -v 7 -g 3 -m 250 -x 500	846	84.04	82.26	[0.3843]			[0.0000]
17	SOAP2	-l 40 -v 5 -g 3 -m 250 -x 500	866	84.07	82.34	[0.3541]			[0.0000]
18	SOAP2	-l 40 -v 3 -g 3 -m 250 -x 500	903	83.65	81.98	[0.3119]			[0.0000]
19	SOAP2	-l 28 -v 7 -g 0 -m 250 -x 500		0.00		[0.0000]			[0.0000]
20	SOAP2	-l 28 -v 5 -g 0 -m 250 -x 500		0.00		[0.0000]			[0.0000]
21	SOAP2	-l 28 -v 3 -g 0 -m 250 -x 500		0.00		[0.0000]			[0.0000]
22	SOAP2	-l 28 -v 7 -g 3 -m 250 -x 500		0.00		[0.0000]			[0.0000]
23	SOAP2	-l 28 -v 5 -g 3 -m 250 -x 500		0.00		[0.0000]			[0.0000]
24	SOAP2	-l 28 -v 3 -g 3 -m 250 -x 500		0.00		[0.0000]			[0.0000]

	Aligner	Options	Time	%Aligned	%Unique	[%Err]		%Q10	[%Err]
1	SeqAlto	-f [Index mode: 0, k-mer_size: 28]	1159	99.59	97.04	[0.0864]		96.89	[0.0719]
2	SeqAlto	-a [Index mode: 0, k-mer_size: 28]	2799	99.61	97.16	[0.0865]		97.02	[0.0815]
3	SeqAlto	-f -u [Index mode: 0, k-mer_size: 28]	823	95.73	93.21	[0.1220]		92.95	[0.0890]
4	SeqAlto	-a -u [Index mode: 0, k-mer_size: 28]	826	95.73	93.21	[0.1220]		92.95	[0.0890]
5	SeqAlto	-f [Index mode: 1, k-mer_size: 22]	934	99.57	96.84	[0.0992]		96.69	[0.0782]
6	SeqAlto	-a [Index mode: 1, k-mer_size: 22]	2945	99.61	97.15	[0.0833]		97.02	[0.0788]
7	SeqAlto	-f -u [Index mode: 1, k-mer_size: 22]	544	95.63	93.07	[0.1387]		92.81	[0.0986]
8	SeqAlto	-a -u [Index mode: 1, k-mer_size: 22]	872	95.86	93.26	[0.1081]		93.02	[0.0920]

Illumina-like 150 bp (paired-end) dataset

	Aligner	Options	Time	%Aligned	%Unique	[%Err]		%Q10	[%Err]
1	HIA	-r 0.011 -x 50 -s 4	443	99.92	98.09	[0.1360]		97.14	[0.0508]

2	HIA	-r 0.011 -x 100 -s 4	482	99.93	98.08	[0.1325]		97.14	[0.0503]
3	HIA	-r 0.011 -x 200 -s 4	482	99.93	98.06	[0.1288]		97.14	[0.0499]
4	HIA	-r 0.011 -x 300 -s 4	545	99.93	98.05	[0.1245]		97.12	[0.0492]
5	HIA	-r 0.021 -x 50 -s 4	521	99.99	98.12	[0.0923]		97.62	[0.0489]
6	HIA	-r 0.021 -x 100 -s 4	566	99.99	98.10	[0.0825]		97.62	[0.0460]
7	HIA	-r 0.021 -x 200 -s 4	701	99.99	98.09	[0.0793]		97.61	[0.0458]
8	HIA	-r 0.021 -x 300 -s 4	722	99.99	98.08	[0.0766]		97.61	[0.0453]
9	HIA	-r 0.031 -x 50 -s 4	668	99.99	98.11	[0.0957]		97.77	[0.0554]
10	HIA	-r 0.031 -x 100 -s 4	696	99.99	98.11	[0.0812]		97.78	[0.0494]
11	HIA	-r 0.031 -x 200 -s 4	856	99.99	98.09	[0.0729]		97.80	[0.0455]
12	HIA	-r 0.031 -x 300 -s 4	1043	99.99	98.08	[0.0690]		97.79	[0.0440]
13	HIA	-r 0.041 -x 50 -s 4	770	99.99	98.12	[0.1133]		97.85	[0.0719]
14	HIA	-r 0.041 -x 100 -s 4	783	99.99	98.12	[0.0954]		97.87	[0.0628]
15	HIA	-r 0.041 -x 200 -s 4	1014	99.99	98.11	[0.0836]		97.88	[0.0568]
16	HIA	-r 0.041 -x 300 -s 4	1153	99.99	98.10	[0.0780]		97.89	[0.0537]

	Aligner	Options	Time	%Aligned	%Unique	[%Err]		%Q10	[%Err]
1	BWA	-k 1 -l 32 -o 1	960	98.53	97.15	[0.3545]		97.04	[0.3419]
2	BWA	-k 1 -l 32 -o 2	1182	99.28	97.79	[0.2726]		97.69	[0.2611]
3	BWA	-k 1 -l 28 -o 1	1208	98.67	97.27	[0.3299]		97.16	[0.3174]
4	BWA	-k 1 -l 32 -o 3	1278	99.33	97.84	[0.2644]		97.73	[0.2525]
5	BWA	-k 1 -l 28 -o 2	1508	99.39	97.88	[0.2458]		97.77	[0.2341]
6	BWA	-k 1 -l 24 -o 1	1598	98.80	97.38	[0.3045]		97.28	[0.2920]
7	BWA	-k 1 -l 28 -o 3	1621	99.44	97.92	[0.2362]		97.81	[0.2243]
8	BWA	-k 1 -l 24 -o 2	1966	99.49	97.96	[0.2154]		97.85	[0.2042]
9	BWA	-k 1 -l 24 -o 3	2095	99.53	97.99	[0.2053]		97.88	[0.1943]
10	BWA	-k 2 -l 32 -o 1	2553	99.12	97.65	[0.2265]		97.55	[0.2142]
11	BWA	-k 2 -l 28 -o 1	3311	99.16	97.69	[0.2182]		97.58	[0.2064]
12	BWA	-k 2 -l 32 -o 2	2983	99.71	98.12	[0.1275]		98.01	[0.1166]
13	BWA	-k 2 -l 32 -o 3	3083	99.74	98.14	[0.1169]		98.03	[0.1058]
14	BWA	-k 2 -l 28 -o 2	3793	99.73	98.13	[0.1177]		98.02	[0.1068]
15	BWA	-k 2 -l 24 -o 1	4315	99.19	97.71	[0.2130]		97.60	[0.2007]

16	BWA	-k 2 -l 28 -o 3	3900	99.76	98.16 [0.1068]		98.05 [0.0960]
17	BWA	-k 2 -l 24 -o 2	4866	99.75	98.15 [0.1092]		98.04 [0.0986]
18	BWA	-k 2 -l 24 -o 3	5380	99.78	98.17 [0.0983]		98.06 [0.0876]

	Aligner	Options	Time	%Aligned	%Unique [%Err]		%Q10 [%Err]
1	BWA MEM	-k 17 -r 1.3	769	99.99	98.43 [0.0140]		98.39 [0.0096]
2	BWA MEM	-k 17 -r 1.5	752	99.99	98.43 [0.0140]		98.38 [0.0096]
3	BWA MEM	-k 17 -r 1.7	741	99.99	98.43 [0.0140]		98.39 [0.0095]
4	BWA MEM	-k 19 -r 1.3	692	99.99	98.43 [0.0138]		98.39 [0.0096]
5	BWA MEM	-k 19 -r 1.5	683	99.99	98.43 [0.0139]		98.39 [0.0096]
6	BWA MEM	-k 19 -r 1.7	671	99.99	98.43 [0.0139]		98.39 [0.0096]
7	BWA MEM	-k 21 -r 1.3	663	99.99	98.43 [0.0139]		98.39 [0.0096]
8	BWA MEM	-k 21 -r 1.5	657	99.99	98.43 [0.0139]		98.39 [0.0096]
9	BWA MEM	-k 21 -r 1.7	649	99.99	98.43 [0.0137]		98.39 [0.0092]

	Aligner	Options	Time	%Aligned	%Unique [%Err]		%Q10 [%Err]
1	Bowtie2	-D 5 -R 1 -N 0 -L 22 -i S,0,2.50	757	96.96	96.90 [1.1354]		93.90 [0.0195]
2	Bowtie2	-D 10 -R 2 -N 0 -L 22 -i S,0,2.50	796	97.01	96.96 [1.1187]		93.95 [0.0147]
3	Bowtie2	-D 15 -R 2 -N 0 -L 22 -i S,1,2.50	901	97.03	97.00 [1.1053]		93.97 [0.0119]
4	Bowtie2	-D 15 -R 2 -N 0 -L 22 -i S,1,2.20	946	97.08	97.04 [1.0819]		94.03 [0.0104]
5	Bowtie2	-D 15 -R 2 -N 0 -L 22 -i S,1,1.65	990	97.10	97.07 [1.0576]		94.07 [0.0117]
6	Bowtie2	-D 15 -R 2 -N 0 -L 22 -i S,1,1.15	1042	97.11	97.08 [1.0366]		94.09 [0.0122]
7	Bowtie2	-D 15 -R 2 -N 0 -L 21 -i S,1,1.00	1120	97.11	97.09 [1.0419]		94.10 [0.0070]
8	Bowtie2	-D 15 -R 2 -N 0 -L 20 -i S,1,0.75	1305	97.11	97.09 [1.0368]		94.10 [0.0104]
9	Bowtie2	-D 15 -R 2 -N 0 -L 20 -i S,1,0.50	1538	97.11	97.09 [1.0254]		94.12 [0.0112]
10	Bowtie2	-D 20 -R 3 -N 0 -L 20 -i S,1,0.50	2174	97.13	97.11 [1.0036]		94.13 [0.0087]
11	Bowtie2	-D 25 -R 4 -N 0 -L 20 -i S,1,0.50	2348	97.13	97.12 [0.9904]		94.14 [0.0083]

	Aligner	Options	Time	%Aligned	%Unique [%Err]		%Q10 [%Err]
1	SOAP2	-l 256 -v 3 -g 0 -m 250 -x 500	380	39.51	38.80 [0.0157]		[0.0000]
2	SOAP2	-l 256 -v 5 -g 0 -m 250 -x 500	379	39.51	38.80 [0.0157]		[0.0000]
3	SOAP2	-l 256 -v 7 -g 0 -m 250 -x 500	380	39.51	38.80 [0.0157]		[0.0000]

4	SOAP2	-l 75 -v 7 -g 0 -m 250 -x 500	505	43.67	42.87	[0.0490]			[0.0000]
5	SOAP2	-l 75 -v 5 -g 0 -m 250 -x 500	506	42.99	42.21	[0.0422]			[0.0000]
6	SOAP2	-l 75 -v 3 -g 0 -m 250 -x 500	505	42.24	41.48	[0.0318]			[0.0000]
7	SOAP2	-l 75 -v 7 -g 3 -m 250 -x 500	856	75.25	74.04	[0.3487]			[0.0000]
8	SOAP2	-l 75 -v 5 -g 3 -m 250 -x 500	865	75.02	73.85	[0.3358]			[0.0000]
9	SOAP2	-l 256 -v 7 -g 3 -m 250 -x 500	771	74.44	73.28	[0.3443]			[0.0000]
10	SOAP2	-l 256 -v 5 -g 3 -m 250 -x 500	779	74.35	73.21	[0.3344]			[0.0000]
11	SOAP2	-l 75 -v 3 -g 3 -m 250 -x 500	876	73.18	72.08	[0.3037]			[0.0000]
12	SOAP2	-l 256 -v 3 -g 3 -m 250 -x 500	767	70.71	69.65	[0.3041]			[0.0000]
13	SOAP2	-l 40 -v 7 -g 0 -m 250 -x 500	1074	44.10	43.27	[0.0654]			[0.0000]
14	SOAP2	-l 40 -v 5 -g 0 -m 250 -x 500	1072	43.39	42.59	[0.0505]			[0.0000]
15	SOAP2	-l 40 -v 3 -g 0 -m 250 -x 500	1097	42.53	41.76	[0.0342]			[0.0000]
16	SOAP2	-l 40 -v 7 -g 3 -m 250 -x 500	1435	75.17	73.95	[0.3612]			[0.0000]
17	SOAP2	-l 40 -v 5 -g 3 -m 250 -x 500	1615	75.01	73.83	[0.3406]			[0.0000]
18	SOAP2	-l 40 -v 3 -g 3 -m 250 -x 500	1460	73.42	72.31	[0.3044]			[0.0000]
19	SOAP2	-l 28 -v 7 -g 0 -m 250 -x 500		0.00		[0.0000]			[0.0000]
20	SOAP2	-l 28 -v 5 -g 0 -m 250 -x 500		0.00		[0.0000]			[0.0000]
21	SOAP2	-l 28 -v 3 -g 0 -m 250 -x 500		0.00		[0.0000]			[0.0000]
22	SOAP2	-l 28 -v 7 -g 3 -m 250 -x 500		0.00		[0.0000]			[0.0000]
23	SOAP2	-l 28 -v 5 -g 3 -m 250 -x 500		0.00		[0.0000]			[0.0000]
24	SOAP2	-l 28 -v 3 -g 3 -m 250 -x 500		0.00		[0.0000]			[0.0000]

	Aligner	Options	Time	%Aligned	%Unique	[%Err]		%Q10	[%Err]
1	SeqAlto	-f [Index mode: 0, k-mer_size: 28]	2053	99.58	97.73	[0.0733]		97.69	[0.0684]
2	SeqAlto	-a [Index mode: 0, k-mer_size: 28]	6877	99.58	97.80	[0.0764]		97.74	[0.0740]
3	SeqAlto	-f -u [Index mode: 0, k-mer_size: 28]	1007	91.53	89.68	[0.1198]		89.54	[0.0957]
4	SeqAlto	-a -u [Index mode: 0, k-mer_size: 28]	1026	91.53	89.68	[0.1198]		89.54	[0.0957]
5	SeqAlto	-f [Index mode: 1, k-mer_size: 22]	1939	99.58	97.65	[0.0783]		97.61	[0.0703]
6	SeqAlto	-a [Index mode: 1, k-mer_size: 22]	7191	99.58	97.80	[0.0723]		97.74	[0.0698]
7	SeqAlto	-f -u [Index mode: 1, k-mer_size: 22]	746	91.49	89.63	[0.1267]		89.50	[0.0968]
8	SeqAlto	-a -u [Index mode: 1, k-mer_size: 22]	992	91.64	89.73	[0.0923]		89.60	[0.0788]

454-like 250 bp (single-end) dataset

	Aligner	Options	Time	%Aligned	%Unique [%Err]		%Q10 [%Err]
1	HIA	-r 0.011 -x 50 -s 4	771	96.49	95.02 [0.4536]		91.96 [0.1187]
2	HIA	-r 0.011 -x 100 -s 4	1009	97.70	96.20 [0.4338]		93.00 [0.1190]
3	HIA	-r 0.011 -x 200 -s 4	1175	98.47	96.95 [0.4349]		93.62 [0.1259]
4	HIA	-r 0.011 -x 300 -s 4	1317	98.81	97.28 [0.4380]		93.87 [0.1288]
5	HIA	-r 0.021 -x 50 -s 4	805	99.81	98.24 [0.5380]		93.84 [0.1419]
6	HIA	-r 0.021 -x 100 -s 4	848	99.89	98.32 [0.5325]		93.81 [0.1467]
7	HIA	-r 0.021 -x 200 -s 4	981	99.92	98.35 [0.5462]		93.72 [0.1547]
8	HIA	-r 0.021 -x 300 -s 4	1087	99.93	98.36 [0.5475]		93.65 [0.1494]
9	HIA	-r 0.031 -x 50 -s 4	692	99.95	98.29 [0.4544]		93.40 [0.1373]
10	HIA	-r 0.031 -x 100 -s 4	818	99.96	98.30 [0.4507]		93.29 [0.1446]
11	HIA	-r 0.031 -x 200 -s 4	800	99.96	98.31 [0.4559]		92.94 [0.1475]
12	HIA	-r 0.031 -x 300 -s 4	917	99.96	98.31 [0.4571]		92.91 [0.1419]
13	HIA	-r 0.041 -x 50 -s 4	833	99.95	98.26 [0.4149]		94.62 [0.1718]
14	HIA	-r 0.041 -x 100 -s 4	856	99.96	98.28 [0.4133]		94.61 [0.1779]
15	HIA	-r 0.041 -x 200 -s 4	955	99.96	98.28 [0.4182]		94.49 [0.1784]
16	HIA	-r 0.041 -x 300 -s 4	1009	99.96	98.28 [0.4189]		94.38 [0.1772]

	Aligner	Options	Time	%Aligned	%Unique [%Err]		%Q10 [%Err]
1	BWA-SW	-c 5.5 -z 1 -s 1	1589	96.85	93.87 [1.7514]		90.46 [0.1199]
2	BWA-SW	-c 5.5 -z 1 -s 2	1727	96.85	94.24 [1.4921]		90.98 [0.0857]
3	BWA-SW	-c 5.5 -z 2 -s 1	2249	99.83	97.46 [0.8630]		94.24 [0.0359]
4	BWA-SW	-c 5.5 -z 1 -s 3	1852	96.85	94.32 [1.3510]		91.18 [0.0780]
5	BWA-SW	-c 5.5 -z 3 -s 1	2629	99.86	97.51 [0.8282]		93.81 [0.0329]
6	BWA-SW	-c 5.5 -z 2 -s 2	2462	99.83	97.52 [0.7380]		94.66 [0.0357]
7	BWA-SW	-c 5.5 -z 2 -s 3	2654	99.83	97.53 [0.6827]		94.79 [0.0363]
8	BWA-SW	-c 5.5 -z 3 -s 2	2908	99.86	97.59 [0.7327]		94.23 [0.0351]
9	BWA-SW	-c 5.5 -z 3 -s 3	3157	99.86	97.61 [0.6735]		94.38 [0.0357]

	Aligner	Options	Time	%Aligned	%Unique [%Err]		%Q10 [%Err]

1	BWA MEM	-k 17 -r 1.3	1497	100.00	97.92	[0.0767]		97.26	[0.0346]
2	BWA MEM	-k 17 -r 1.5	1425	100.00	97.92	[0.0796]		97.26	[0.0362]
3	BWA MEM	-k 17 -r 1.7	1353	100.00	97.92	[0.0838]		97.25	[0.0381]
4	BWA MEM	-k 19 -r 1.3	1176	100.00	97.92	[0.0801]		97.25	[0.0372]
5	BWA MEM	-k 19 -r 1.5	1133	100.00	97.91	[0.0846]		97.25	[0.0385]
6	BWA MEM	-k 19 -r 1.7	1095	100.00	97.91	[0.0897]		97.24	[0.0395]
7	BWA MEM	-k 21 -r 1.3	1096	100.00	97.91	[0.0856]		97.24	[0.0383]
8	BWA MEM	-k 21 -r 1.5	1045	100.00	97.90	[0.0921]		97.23	[0.0412]
9	BWA MEM	-k 21 -r 1.7	990	100.00	97.89	[0.1039]		97.22	[0.0460]

	Aligner	Options	Time	%Aligned	%Unique	[%Err]		%Q10	[%Err]
1	Bowtie2	-D 5 -R 1 -N 0 -L 25 -i S,1,2.0	413	90.32	78.23	[1.5838]		35.87	[0.0084]
2	Bowtie2	-D 5 -R 1 -N 0 -L 22 -i S,1,2.50	418	88.72	76.58	[1.7353]		35.19	[0.0136]
3	Bowtie2	-D 10 -R 2 -N 0 -L 22 -i S,1,2.50	608	91.24	78.62	[1.4714]		35.90	[0.0061]
4	Bowtie2	-D 10 -R 2 -N 0 -L 22 -i S,1,1.75	706	95.88	81.67	[1.2559]		36.39	[0.0066]
5	Bowtie2	-D 15 -R 2 -N 0 -L 22 -i S,1,2.50	728	91.65	78.90	[1.3712]		35.97	[0.0050]
6	Bowtie2	-D 15 -R 2 -N 0 -L 22 -i S,1,2.20	772	93.65	80.28	[1.2681]		36.21	[0.0039]
7	Bowtie2	-D 15 -R 2 -N 0 -L 22 -i S,1,1.65	867	96.72	82.15	[1.1105]		36.44	[0.0022]
8	Bowtie2	-D 15 -R 2 -N 0 -L 22 -i S,1,1.15	989	98.40	82.93	[0.9650]		36.49	[0.0025]
9	Bowtie2	-D 15 -R 2 -N 0 -L 21 -i S,1,1.00	1109	98.76	83.05	[0.9504]		36.49	[0.0016]
10	Bowtie2	-D 15 -R 2 -N 0 -L 20 -i S,1,0.75	1359	99.06	83.13	[0.9106]		36.47	[0.0016]
11	Bowtie2	-D 15 -R 2 -N 0 -L 20 -i S,1,0.50	1591	99.24	83.23	[0.8648]		36.49	[0.0014]
12	Bowtie2	-D 20 -R 3 -N 0 -L 20 -i S,1,0.50	2224	99.47	83.35	[0.7182]		36.47	[0.0011]
13	Bowtie2	-D 25 -R 4 -N 0 -L 20 -i S,1,0.50	2947	99.59	83.40	[0.5980]		36.44	[0.0011]

454-like 400 bp (single-end) dataset

	Aligner	Options	Time	%Aligned	%Unique	[%Err]		%Q10	[%Err]
1	HIA	-r 0.011 -x 50 -s 4	1499	97.75	96.60	[0.2642]		93.94	[0.0379]
2	HIA	-r 0.011 -x 100 -s 4	1856	98.63	97.45	[0.2475]		94.71	[0.0348]
3	HIA	-r 0.011 -x 200 -s 4	2518	99.09	97.91	[0.2428]		95.11	[0.0367]
4	HIA	-r 0.011 -x 300 -s 4	2582	99.26	98.08	[0.2439]		95.26	[0.0393]

5	HIA	-r 0.021 -x 50 -s 4	1139	99.74	98.57	[0.2681]		95.50	[0.0269]
6	HIA	-r 0.021 -x 100 -s 4	1256	99.75	98.58	[0.2646]		95.51	[0.0284]
7	HIA	-r 0.021 -x 200 -s 4	1377	99.76	98.59	[0.2654]		95.49	[0.0302]
8	HIA	-r 0.021 -x 300 -s 4	1495	99.76	98.59	[0.2686]		95.48	[0.0333]
9	HIA	-r 0.031 -x 50 -s 4	958	99.75	98.51	[0.1938]		95.34	[0.0228]
10	HIA	-r 0.031 -x 100 -s 4	1021	99.76	98.52	[0.1865]		95.36	[0.0220]
11	HIA	-r 0.031 -x 200 -s 4	1016	99.76	98.52	[0.1869]		95.37	[0.0233]
12	HIA	-r 0.031 -x 300 -s 4	1135	99.76	98.52	[0.1861]		95.36	[0.0251]
13	HIA	-r 0.041 -x 50 -s 4	1076	99.75	98.47	[0.1678]		96.09	[0.0398]
14	HIA	-r 0.041 -x 100 -s 4	1199	99.76	98.48	[0.1574]		96.13	[0.0376]
15	HIA	-r 0.041 -x 200 -s 4	1319	99.76	98.48	[0.1539]		96.16	[0.0382]
16	HIA	-r 0.041 -x 300 -s 4	1378	99.76	98.48	[0.1557]		96.17	[0.0397]

	Aligner	Options	Time	%Aligned	%Unique	[%Err]		%Q10	[%Err]
1	BWA-SW	-c 5.5 -z 1 -s 1	2767	99.25	92.51	[0.9058]		90.29	[0.0807]
2	BWA-SW	-c 5.5 -z 1 -s 2	3009	99.25	92.82	[0.6624]		90.72	[0.0572]
3	BWA-SW	-c 5.5 -z 2 -s 1	3327	99.99	94.74	[0.2990]		92.81	[0.0288]
4	BWA-SW	-c 5.5 -z 1 -s 3	3191	99.25	92.90	[0.5512]		90.87	[0.0489]
5	BWA-SW	-c 5.5 -z 3 -s 1	4142	100.00	95.76	[0.2871]		93.66	[0.0279]
6	BWA-SW	-c 5.5 -z 2 -s 2	3721	99.99	94.85	[0.2349]		93.06	[0.0279]
7	BWA-SW	-c 5.5 -z 2 -s 3	4070	99.99	94.89	[0.2132]		93.15	[0.0293]
8	BWA-SW	-c 5.5 -z 3 -s 2	4666	100.00	95.86	[0.2273]		93.91	[0.0279]
9	BWA-SW	-c 5.5 -z 3 -s 3	5144	100.00	95.89	[0.2084]		94.00	[0.0284]

	Aligner	Options	Time	%Aligned	%Unique	[%Err]		%Q10	[%Err]
1	BWA MEM	-k 17 -r 1.3	2426	99.99	98.46	[0.0471]		97.98	[0.0238]
2	BWA MEM	-k 17 -r 1.5	2302	100.00	98.47	[0.0492]		97.99	[0.0250]
3	BWA MEM	-k 17 -r 1.7	2199	100.00	98.46	[0.0505]		97.98	[0.0252]
4	BWA MEM	-k 19 -r 1.3	1906	100.00	98.47	[0.0485]		97.99	[0.0246]
5	BWA MEM	-k 19 -r 1.5	1851	100.00	98.47	[0.0496]		97.99	[0.0248]
6	BWA MEM	-k 19 -r 1.7	1780	100.00	98.47	[0.0518]		97.98	[0.0256]
7	BWA MEM	-k 21 -r 1.3	1771	100.00	98.47	[0.0489]		97.98	[0.0248]

8	BWA MEM	-k 21 -r 1.5	1714	100.00	98.46	[0.0505]		97.98	[0.0249]
9	BWA MEM	-k 21 -r 1.7	1643	100.00	98.46	[0.0530]		97.98	[0.0255]

	Aligner	Options	Time	%Aligned	%Unique	[%Err]		%Q10	[%Err]
1	Bowtie2	-D 5 -R 1 -N 0 -L 25 -i S,1,2.0	843	94.89	85.27	[1.1837]		32.70	[0.0040]
2	Bowtie2	-D 5 -R 1 -N 0 -L 22 -i S,1,2.50	900	93.57	84.05	[1.3152]		32.33	[0.0056]
3	Bowtie2	-D 10 -R 2 -N 0 -L 22 -i S,1,2.50	1380	95.77	85.80	[1.0281]		32.77	[0.0018]
4	Bowtie2	-D 10 -R 2 -N 0 -L 22 -i S,1,1.75	1615	98.49	87.69	[0.8286]		32.98	[0.0006]
5	Bowtie2	-D 15 -R 2 -N 0 -L 22 -i S,1,2.50	1736	96.03	85.96	[0.9282]		32.79	[0.0037]
6	Bowtie2	-D 15 -R 2 -N 0 -L 22 -i S,1,2.20	1799	97.26	86.84	[0.8402]		32.91	[0.0018]
7	Bowtie2	-D 15 -R 2 -N 0 -L 22 -i S,1,1.65	1980	98.90	87.89	[0.7075]		32.98	[0.0009]
8	Bowtie2	-D 15 -R 2 -N 0 -L 22 -i S,1,1.15	2217	99.64	88.28	[0.5852]		32.99	[0.0003]
9	Bowtie2	-D 15 -R 2 -N 0 -L 21 -i S,1,1.00	2463	99.76	88.34	[0.5595]		32.98	[0.0006]
10	Bowtie2	-D 15 -R 2 -N 0 -L 20 -i S,1,0.75	2941	99.83	88.37	[0.5474]		32.97	[0.0000]
11	Bowtie2	-D 15 -R 2 -N 0 -L 20 -i S,1,0.50	3363	99.88	88.38	[0.5061]		32.97	[0.0003]
12	Bowtie2	-D 20 -R 3 -N 0 -L 20 -i S,1,0.50	4860	99.93	88.36	[0.3818]		32.95	[0.0000]
13	Bowtie2	-D 25 -R 4 -N 0 -L 20 -i S,1,0.50	6597	99.96	88.35	[0.3048]		32.93	[0.0000]

10. Results for real datasets

The following are the results for real datasets. Time measure is elapsed time (second). Unique refers to MAPQ ≥ 1 if MAPQ is reported. Q10 refers to MAPQ ≥ 10.

For HiSeq 101 bp (single-end) dataset and 454-like 400 bp (single-end), HIA and BWA MEM are higher ranks than the other aligners in terms of the speed and the total number of reads aligned. For the paired-end reads, the aligned percentage of Bowtie2 is higher than the other aligners, but HIA is faster than all of the other aligners except SOAP2.

HiSeq 101 bp (single-end) dataset

	Aligner	Options	Time	%Aligned	%Unique	%Q10
1	HIA	-r 0.011 -x 50 -s 4	252	90.37	86.08	82.94
2	HIA	-r 0.011 -x 100 -s 4	288	90.55	86.19	82.93
3	HIA	-r 0.011 -x 200 -s 4	328	90.73	86.30	82.95
4	HIA	-r 0.011 -x 300 -s 4	359	90.84	86.38	82.96

5	HIA	-r 0.021 -x 50 -s 4	262	94.30	89.34	85.28
6	HIA	-r 0.021 -x 100 -s 4	301	94.46	89.42	85.21
7	HIA	-r 0.021 -x 200 -s 4	356	94.57	89.46	85.12
8	HIA	-r 0.021 -x 300 -s 4	368	94.61	89.48	85.08
9	HIA	-r 0.031 -x 50 -s 4	314	96.30	90.99	86.29
10	HIA	-r 0.031 -x 100 -s 4	381	96.32	90.90	86.08
11	HIA	-r 0.031 -x 200 -s 4	439	96.33	90.82	85.87
12	HIA	-r 0.031 -x 300 -s 4	458	96.34	90.78	85.73
13	HIA	-r 0.041 -x 50 -s 4	345	96.69	91.32	86.65
14	HIA	-r 0.041 -x 100 -s 4	369	97.71	91.23	86.41
15	HIA	-r 0.041 -x 200 -s 4	433	96.72	91.11	86.20
16	HIA	-r 0.041 -x 300 -s 4	498	96.73	91.06	86.09

	Aligner	Options	Time	%Aligned	%Unique	%Q10
1	BWA	-k 1 -l 32 -o 1	547	85.16	81.27	81.14
2	BWA	-k 1 -l 32 -o 2	788	85.21	81.32	81.19
3	BWA	-k 1 -l 28 -o 1	683	85.32	81.40	81.27
4	BWA	-k 1 -l 32 -o 3	915	85.22	81.33	81.20
5	BWA	-k 1 -l 28 -o 2	958	85.37	81.45	81.32
6	BWA	-k 1 -l 24 -o 1	876	85.46	81.51	81.38
7	BWA	-k 1 -l 28 -o 3	1127	85.38	81.45	81.32
8	BWA	-k 1 -l 24 -o 2	1212	85.51	81.56	81.43
9	BWA	-k 1 -l 24 -o 3	1419	85.52	81.56	81.43
10	BWA	-k 2 -l 32 -o 1	1702	85.74	81.71	81.57
11	BWA	-k 2 -l 28 -o 1	1944	85.78	81.74	81.60
12	BWA	-k 2 -l 32 -o 2	2081	85.79	81.76	81.63
13	BWA	-k 2 -l 32 -o 3	2284	85.79	81.76	81.63
14	BWA	-k 2 -l 28 -o 2	2440	85.83	81.79	81.65
15	BWA	-k 2 -l 24 -o 1	2296	85.81	81.76	81.63
16	BWA	-k 2 -l 28 -o 3	2668	85.83	81.79	81.66
17	BWA	-k 2 -l 24 -o 2	2877	85.87	81.82	81.68
18	BWA	-k 2 -l 24 -o 3	3126	85.87	81.82	81.68

	Aligner	Options	Time	%Aligned	%Unique	%Q10
1	BWA MEM	-k 17 -r 1.3	272	96.86	89.32	86.85
2	BWA MEM	-k 17 -r 1.5	272	96.84	89.32	86.86
3	BWA MEM	-k 17 -r 1.7	247	96.82	89.40	86.93
4	BWA MEM	-k 19 -r 1.3	229	96.71	89.21	86.76
5	BWA MEM	-k 19 -r 1.5	224	96.69	89.27	86.81
6	BWA MEM	-k 19 -r 1.7	212	96.68	89.36	86.88
7	BWA MEM	-k 21 -r 1.3	217	96.46	89.07	86.64
8	BWA MEM	-k 21 -r 1.5	204	96.44	89.15	86.72
9	BWA MEM	-k 21 -r 1.7	191	96.44	89.27	86.84

	Aligner	Options	Time	%Aligned	%Unique	%Q10
1	Bowtie2	-D 5 -R 1 -N 0 -L 22 -i S,0,2.50	220	92.43	90.14	82.76
2	Bowtie2	-D 10 -R 2 -N 0 -L 22 -i S,0,2.50	306	93.21	90.82	83.06
3	Bowtie2	-D 15 -R 2 -N 0 -L 22 -i S,1,2.50	346	93.36	90.93	83.10
4	Bowtie2	-D 15 -R 2 -N 0 -L 22 -i S,1,2.20	346	93.44	91.01	83.14
5	Bowtie2	-D 15 -R 2 -N 0 -L 22 -i S,1,1.65	386	93.69	91.20	83.28
6	Bowtie2	-D 15 -R 2 -N 0 -L 22 -i S,1,1.15	439	94.05	91.49	83.45
7	Bowtie2	-D 15 -R 2 -N 0 -L 21 -i S,1,1.00	497	94.27	91.62	83.49
8	Bowtie2	-D 15 -R 2 -N 0 -L 20 -i S,1,0.75	636	94.56	91.80	83.59
9	Bowtie2	-D 15 -R 2 -N 0 -L 20 -i S,1,0.50	727	94.66	91.87	83.63
10	Bowtie2	-D 20 -R 3 -N 0 -L 20 -i S,1,0.50	999	94.85	92.04	83.68
11	Bowtie2	-D 25 -R 4 -N 0 -L 20 -i S,1,0.50	1291	94.96	92.11	83.69

	Aligner	Options	Time	%Aligned	%Unique	%Q10
1	SOAP2	-l 256 -v 3 -g 0	159	77.15	74.05	
2	SOAP2	-l 256 -v 5 -g 0	155	77.15	74.05	
3	SOAP2	-l 256 -v 7 -g 0	155	77.15	74.05	
4	SOAP2	-l 75 -v 5 -g 0	194	84.35	80.51	
5	SOAP2	-l 75 -v 7 -g 0	192	85.27	81.33	
6	SOAP2	-l 75 -v 3 -g 0	202	82.24	78.60	

7	SOAP2	-l 40 -v 7 -g 0	283	87.29	82.29	
8	SOAP2	-l 40 -v 5 -g 0	314	85.23	80.83	
9	SOAP2	-l 40 -v 3 -g 0	367	81.77	78.07	
10	SOAP2	-l 28-v 7 -g 0	1018	87.63	82.30	
11	SOAP2	-l 28 -v 5 -g 0	1160	85.28	80.78	
12	SOAP2	-l 28 -v 3 -g 0	1396	81.59	77.93	

	Aligner	Options	Time	%Aligned	%Unique	%Q10
1	SeqAlto	-f [Index mode: 0, k-mer_size: 28]	598	88.66	85.24	84.65
2	SeqAlto	-a [Index mode: 0, k-mer_size: 28]	1461	88.72	85.08	84.46
3	SeqAlto	-f -u [Index mode: 0, k-mer_size: 28]	391	86.08	82.74	82.27
4	SeqAlto	-a -u [Index mode: 0, k-mer_size: 28]	455	86.19	82.64	82.14
5	SeqAlto	-f [Index mode: 1, k-mer_size: 22]	382	88.78	85.23	84.63
6	SeqAlto	-a [Index mode: 1, k-mer_size: 22]	1567	89.16	85.22	84.60
7	SeqAlto	-f -u [Index mode: 1, k-mer_size: 22]	128	86.15	82.71	82.22
8	SeqAlto	-a -u [Index mode: 1, k-mer_size: 22]	198	86.53	82.27	82.22

454-like 400 bp (single-end)

	Aligner	Options	Time	%Aligned	%Unique	%Q10
1	HIA	-r 0.011 -x 50 -s 4	441	97.47	95.83	93.30
2	HIA	-r 0.011 -x 100 -s 4	453	99.05	95.88	93.27
3	HIA	-r 0.011 -x 200 -s 4	498	97.61	95.91	93.22
4	HIA	-r 0.011 -x 300 -s 4	528	97.64	95.93	93.20
5	HIA	-r 0.021 -x 50 -s 4	460	98.42	96.52	94.80
6	HIA	-r 0.021 -x 100 -s 4	546	98.48	96.56	94.78
7	HIA	-r 0.021 -x 200 -s 4	622	98.54	96.59	94.75
8	HIA	-r 0.021 -x 300 -s 4	693	98.56	96.60	94.72
9	HIA	-r 0.031 -x 50 -s 4	498	98.96	96.91	95.70
10	HIA	-r 0.031 -x 100 -s 4	597	98.97	96.90	95.67
11	HIA	-r 0.031 -x 200 -s 4	664	98.98	96.89	95.63
12	HIA	-r 0.031 -x 300 -s 4	815	98.98	96.88	95.61

13	HIA	-r 0.041 -x 50 -s 4	622	99.04	96.95	96.02
14	HIA	-r 0.041 -x 100 -s 4	749	99.04	96.95	96.00
15	HIA	-r 0.041 -x 200 -s 4	823	99.04	96.91	95.95
16	HIA	-r 0.041 -x 300 -s 4	964	99.05	96.90	95.92

	Aligner	Options	Time	%Aligned	%Unique	%Q10
1	BWA-SW	-c 5.5 -z 1 -s 1	2081	99.20	95.86	92.35
2	BWA-SW	-c 5.5 -z 1 -s 2	2708	99.23	95.97	92.55
3	BWA-SW	-c 5.5 -z 2 -s 1	2913	99.13	96.13	92.23
4	BWA-SW	-c 5.5 -z 1 -s 3	3328	99.20	96.01	92.64
5	BWA-SW	-c 5.5 -z 3 -s 1	3645	99.53	96.32	92.20
6	BWA-SW	-c 5.5 -z 2 -s 2	3947	99.48	96.27	92.50
7	BWA-SW	-c 5.5 -z 2 -s 3	5030	99.48	96.31	92.62
8	BWA-SW	-c 5.5 -z 3 -s 2	5039	99.53	96.44	92.51
9	BWA-SW	-c 5.5 -z 3 -s 3	6369	99.53	96.48	92.63

	Aligner	Options	Time	%Aligned	%Unique	%Q10
1	BWA MEM	-k 17 -r 1.3	851	99.73	96.35	94.85
2	BWA MEM	-k 17 -r 1.5	830	99.73	96.36	94.86
3	BWA MEM	-k 17 -r 1.7	802	99.72	96.40	94.90
4	BWA MEM	-k 19 -r 1.3	742	99.72	96.36	94.87
5	BWA MEM	-k 19 -r 1.5	739	99.72	96.38	94.89
6	BWA MEM	-k 19 -r 1.7	718	99.72	96.42	94.92
7	BWA MEM	-k 21 -r 1.3	708	99.71	96.38	94.90
8	BWA MEM	-k 21 -r 1.5	704	99.70	96.42	94.93
9	BWA MEM	-k 21 -r 1.7	685	99.70	96.47	94.97

	Aligner	Options	Time	%Aligned	%Unique	%Q10
1	Bowtie2	-D 5 -R 1 -N 0 -L 25 -i S,1,2.0	843	97.45	96.18	90.54
2	Bowtie2	-D 5 -R 1 -N 0 -L 22 -i S,1,2.50	900	97.00	95.66	89.96
3	Bowtie2	-D 10 -R 2 -N 0 -L 22 -i S,1,2.50	1380	97.75	96.47	90.72
4	Bowtie2	-D 10 -R 2 -N 0 -L 22 -i S,1,1.75	1615	97.97	96.65	90.88

5	Bowtie2	-D 15 -R 2 -N 0 -L 22 -i S,1,2.50	1736	97.85	96.57	90.78
6	Bowtie2	-D 15 -R 2 -N 0 -L 22 -i S,1,2.20	1799	97.93	96.64	90.83
7	Bowtie2	-D 15 -R 2 -N 0 -L 22 -i S,1,1.65	1980	98.08	96.74	90.93
8	Bowtie2	-D 15 -R 2 -N 0 -L 22 -i S,1,1.15	2217	98.18	96.81	90.97
9	Bowtie2	-D 15 -R 2 -N 0 -L 21 -i S,1,1.00	2463	98.21	96.83	90.97
10	Bowtie2	-D 15 -R 2 -N 0 -L 20 -i S,1,0.75	2941	98.22	96.83	90.97
11	Bowtie2	-D 15 -R 2 -N 0 -L 20 -i S,1,0.50	3363	98.24	96.84	91.01
12	Bowtie2	-D 20 -R 3 -N 0 -L 20 -i S,1,0.50	4860	98.33	96.92	91.02
13	Bowtie2	-D 25 -R 4 -N 0 -L 20 -i S,1,0.50	6597	98.37	96.96	91.02

HiSeq 101bp×99 bp (paired-end) dataset

	Aligner	Options	Time	%Aligned	%Unique	%Q10
1	HIA	-r 0.011 -x 50 -s 4	419	82.84	79.81	78.64
2	HIA	-r 0.011 -x 100 -s 4	480	82.95	79.87	78.69
3	HIA	-r 0.011 -x 200 -s 4	596	83.01	79.89	78.70
4	HIA	-r 0.011 -x 300 -s 4	629	83.04	79.89	78.71
5	HIA	-r 0.021 -x 50 -s 4	466	87.27	83.92	82.40
6	HIA	-r 0.021 -x 100 -s 4	524	87.48	84.06	82.51
7	HIA	-r 0.021 -x 200 -s 4	643	87.62	84.15	82.55
8	HIA	-r 0.021 -x 300 -s 4	675	87.66	84.17	82.57
9	HIA	-r 0.031 -x 50 -s 4	684	89.78	86.10	84.06
10	HIA	-r 0.031 -x 100 -s 4	726	89.95	86.19	84.07
11	HIA	-r 0.031 -x 200 -s 4	822	90.16	86.31	84.08
12	HIA	-r 0.031 -x 300 -s 4	935	90.30	86.40	84.09
13	HIA	-r 0.041 -x 50 -s 4	734	90.93	87.13	85.24
14	HIA	-r 0.041 -x 100 -s 4	855	91.18	87.29	85.27
15	HIA	-r 0.041 -x 200 -s 4	1003	91.39	87.39	85.28
16	HIA	-r 0.041 -x 300 -s 4	1111	91.53	87.48	85.25

	Aligner	Options	Time	%Aligned	%Unique	%Q10
1	BWA	-k 1 -l 32 -o 1	557	88.38	86.19	85.90

2	BWA	-k 1 -l 32 -o 2	761	88.41	86.21	85.93
3	BWA	-k 1 -l 28 -o 1	682	88.50	86.29	85.98
4	BWA	-k 1 -l 32 -o 3	913	88.41	86.22	85.93
5	BWA	-k 1 -l 28 -o 2	954	88.53	86.31	86.01
6	BWA	-k 1 -l 24 -o 1	875	88.62	86.37	86.07
7	BWA	-k 1 -l 28 -o 3	1126	88.53	86.31	86.01
8	BWA	-k 1 -l 24 -o 2	1209	88.65	86.40	86.09
9	BWA	-k 1 -l 24 -o 3	1407	88.65	86.40	86.09
10	BWA	-k 2 -l 32 -o 1	1651	88.65	86.52	86.20
11	BWA	-k 2 -l 28 -o 1	1942	88.84	86.54	86.21
12	BWA	-k 2 -l 32 -o 2	2076	88.85	86.55	86.22
13	BWA	-k 2 -l 32 -o 3	2276	88.85	86.55	86.22
14	BWA	-k 2 -l 28 -o 2	2430	88.87	86.57	86.24
15	BWA	-k 2 -l 24 -o 1	2317	88.87	86.57	86.23
16	BWA	-k 2 -l 28 -o 3	2672	88.88	86.56	86.24
17	BWA	-k 2 -l 24 -o 2	2871	88.90	86.59	86.26
18	BWA	-k 2 -l 24 -o 3	3130	88.90	86.59	86.26

	Aligner	Options	Time	%Aligned	%Unique	%Q10
1	BWA MEM	-k 17 -r 1.3	728	93.49	90.54	89.79
2	BWA MEM	-k 17 -r 1.5	690	93.49	90.55	89.80
3	BWA MEM	-k 17 -r 1.7	659	93.47	90.56	89.83
4	BWA MEM	-k 19 -r 1.3	592	93.19	90.28	89.55
5	BWA MEM	-k 19 -r 1.5	568	93.18	90.29	89.57
6	BWA MEM	-k 19 -r 1.7	546	93.16	90.30	89.58
7	BWA MEM	-k 21 -r 1.3	545	92.88	90.01	89.32
8	BWA MEM	-k 21 -r 1.5	523	92.87	90.02	89.33
9	BWA MEM	-k 21 -r 1.7	495	92.82	90.01	89.33

	Aligner	Options	Time	%Aligned	%Unique	%Q10
1	Bowtie2	-D 5 -R 1 -N 0 -L 22 -i S,0,2.50	530	91.03	90.08	84.44
2	Bowtie2	-D 10 -R 2 -N 0 -L 22 -i S,0,2.50	577	91.27	90.23	84.40

3	Bowtie2	-D 15 -R 2 -N 0 -L 22 -i S,1,2.50	654	91.42	90.31	84.38
4	Bowtie2	-D 15 -R 2 -N 0 -L 22 -i S,1,2.20	657	91.46	90.37	84.41
5	Bowtie2	-D 15 -R 2 -N 0 -L 22 -i S,1,1.65	704	91.82	90.62	84.53
6	Bowtie2	-D 15 -R 2 -N 0 -L 22 -i S,1,1.15	747	92.12	90.83	84.64
7	Bowtie2	-D 15 -R 2 -N 0 -L 21 -i S,1,1.00	845	92.32	90.99	84.70
8	Bowtie2	-D 15 -R 2 -N 0 -L 20 -i S,1,0.75	938	92.54	91.17	84.81
9	Bowtie2	-D 15 -R 2 -N 0 -L 20 -i S,1,0.50	1041	92.63	91.23	84.84
10	Bowtie2	-D 20 -R 3 -N 0 -L 20 -i S,1,0.50	1545	93.03	91.54	84.94
11	Bowtie2	-D 25 -R 4 -N 0 -L 20 -i S,1,0.50	1646	93.13	91.60	84.93

	Aligner	Options	Time	%Aligned	%Unique	%Q10
1	SOAP2	-l 256 -v 3 -g 0 -m 250 -x 500	297	64.30	62.62	
2	SOAP2	-l 256 -v 5 -g 0 -m 250 -x 500	292	64.30	62.62	
3	SOAP2	-l 256 -v 7 -g 0 -m 250 -x 500	292	64.30	62.62	
4	SOAP2	-l 75 -v 7 -g 0 -m 250 -x 500	349	74.97	72.87	
5	SOAP2	-l 75 -v 5 -g 0 -m 250 -x 500	349	73.64	71.58	
6	SOAP2	-l 75 -v 3 -g 0 -m 250 -x 500	352	70.50	68.55	
7	SOAP2	-l 75 -v 7 -g 3 -m 250 -x 500	427	81.45	79.06	
8	SOAP2	-l 75 -v 5 -g 3 -m 250 -x 500	435	78.74	76.52	
9	SOAP2	-l 256 -v 7 -g 3 -m 250 -x 500	431	79.30	77.12	
10	SOAP2	-l 256 -v 5 -g 3 -m 250 -x 500	423	76.57	74.54	
11	SOAP2	-l 75 -v 3 -g 3 -m 250 -x 500	447	74.10	72.09	
12	SOAP2	-l 256 -v 3 -g 3 -m 250 -x 500	425	70.63	68.82	
13	SOAP2	-l 40 -v 7 -g 0 -m 250 -x 500	613	76.38	74.08	
14	SOAP2	-l 40 -v 5 -g 0 -m 250 -x 500	655	73.99	71.82	
15	SOAP2	-l 40 -v 3 -g 0 -m 250 -x 500	726	69.64	67.70	
16	SOAP2	-l 40 -v 7 -g 3 -m 250 -x 500	725	82.18	79.56	
17	SOAP2	-l 40 -v 5 -g 3 -m 250 -x 500	751	78.92	76.58	
18	SOAP2	-l 40 -v 3 -g 3 -m 250 -x 500	825	73.38	71.37	
19	SOAP2	-l 28 -v 7 -g 0 -m 250 -x 500	2247	76.44	74.09	
20	SOAP2	-l 28 -v 5 -g 0 -m 250 -x 500	2447	73.95	71.75	
21	SOAP2	-l 28 -v 3 -g 0 -m 250 -x 500	2796	69.45	67.52	

22	SOAP2	-l 28 -v 7 -g 3 -m 250 -x 500	2390	82.24	79.53	
23	SOAP2	-l 28 -v 5 -g 3 -m 250 -x 500	2554	78.88	76.50	
24	SOAP2	-l 28 -v 3 -g 3 -m 250 -x 500	3342	73.20	71.20	

	Aligner	Options	Time	%Aligned	%Unique	%Q10
1	SeqAlto	-f [Index mode: 0, k-mer_size: 28]	1164	91.55	87.33	87.03
2	SeqAlto	-a [Index mode: 0, k-mer_size: 28]	2873	91.66	87.48	87.18
3	SeqAlto	-f -u [Index mode: 0, k-mer_size: 28]	748	90.20	86.56	86.04
4	SeqAlto	-a -u [Index mode: 0, k-mer_size: 28]	748	90.20	86.56	86.04
5	SeqAlto	-f [Index mode: 1, k-mer_size: 22]	967	91.82	87.55	87.28
6	SeqAlto	-a [Index mode: 1, k-mer_size: 22]	3370	92.04	87.82	87.55
7	SeqAlto	-f -u [Index mode: 1, k-mer_size: 22]	460	90.29	86.59	86.07
8	SeqAlto	-a -u [Index mode: 1, k-mer_size: 22]	672	90.51	86.74	86.21
